# The Proteomic Response of *Arabidopsis thaliana* to Cadmium Sulfide Quantum Dots, and Its Correlation with the Transcriptomic Response

**DOI:** 10.3389/fpls.2015.01104

**Published:** 2015-12-16

**Authors:** Marta Marmiroli, Davide Imperiale, Luca Pagano, Marco Villani, Andrea Zappettini, Nelson Marmiroli

**Affiliations:** ^1^Department of Life Sciences, University of ParmaParma, Italy; ^2^Institute of Materials for Electronics and Magnetism (IMEM-CNR)Parma, Italy

**Keywords:** tolerant mutants, engineered nanomaterials (ENM), genotoxicology ecotoxicology, exposure markers, comparative analysis

## Abstract

A fuller understanding of the interaction between plants and engineered nanomaterials is of topical relevance because the latter are beginning to find applications in agriculture and the food industry. There is a growing need to establish objective safety criteria for their use. The recognition of two independent *Arabidopsis thaliana* mutants displaying a greater level of tolerance than the wild type plant to exposure to cadmium sulfide quantum dots (CdS QDs) has offered the opportunity to characterize the tolerance response at the physiological, transcriptomic, and proteomic levels. Here, a proteomics-based comparison confirmed the conclusions drawn from an earlier transcriptomic analysis that the two mutants responded to CdS QD exposure differently both to the wild type and to each other. Just over half of the proteomic changes mirrored documented changes at the level of gene transcription, but a substantial number of transcript/gene product pairs were altered in the opposite direction. An interpretation of the discrepancies is given, along with some considerations regarding the use and significance of -omics when monitoring the potential toxicity of ENMs for health and environment.

## Introduction

Nanotechnology is regarded as transformative, but its potential long term impact on human health and the environment remains inadequately researched (Colvin, 2003; Royal Society and Royal Academy of Engineering, 2004). Legislative authorities still suffer from a paucity of appropriate data to enable a science-based regulatory framework to be constructed over the release and commercialization of nanomaterials. While much of the focus of nanotechnology has been in the electronics industry and medical research and development, a range of potential applications in agriculture is now opening up, including the incorporation of nanoparticles (NPs) in pesticide formulations, their use as biosensors and devices to aid genetic manipulation and as aids to post-harvest management (Singh Sekhon, 2014; Servin et al., 2015). A wealth of literature over the last decade has addressed the potential toxicity of NPs and enhanced manufactured nanomaterials [ENMs; European Parliament, 2011; Science and Technology Options Assessment (STOA) European Parliament, 2012].

The EU Regulation 1169/2011 (to be applied in December 2016), although attempting a formal definition of ENMs, fails to mention them in the context of food additives, and even the proposed definitions are controversial. Concerns regarding the adequacy of the regulation have been raised by other EU organs [Aschberger et al., 2014; Science and Technology Options Assessment (STOA) European Parliament, 2012].

A general lack of consumer information has been criticized by some parties Friends of the Earth: emerging Tech Project website, 2015 (https://www.foe.org.au/articles/2015-09-25/new-study-raises-further-questions-about-safety-nano-ingredients-food) as has the approach for assessing toxicity and ecotoxicity (Fadeel and Garcia-Bennett, 2010; Saez et al., 2010; Sigg et al., 2014). In a recent report the OECD emphasized the importance of improved toxicity test for assessing ENMs environmental dispersion and impact on human health [Organisation for Economic Co-operation and Development (OECD), 2014]. The potential risks arising from a lack of legislation have been flagged by Abbott et al. (2012) and Hodge et al. (2014). The consensus regarding ENMs is that hard data are still required to clarify the nature and implications of their interaction with biological matrices. Meanwhile, some methods aimed at improving the performance and reducing the toxicity of medical NPs, such as incorporating biocompatible coating materials, modifying their surface to mitigate toxicity and building in biodegradability have been proposed.

Quantum dots (QDs) are crystalline NPs, first synthesized in the early 1980s for use in the electronics industry (Brus, 1984). Cadmium sulfide quantum dots (CdS QDs) have a high surface charge and reactivity and are very stable (Favero et al., 2006). Their biological activity has been studied using both a plant and a yeast model (Marmiroli et al., 2014, 2015), applying both a mutant-based and a genome-wide transcriptomics approach. Two *Arabidopsis thaliana* mutants have emerged as showing an enhanced level of tolerance; in the first, the mutagenized genes encoded an unknown chloroplast-localized protein, a cytoplasm-localized calmodulin binding protein and a member of the MYB class of transcription factors, while in the second, the candidate genes encoded an O-glycosyl hydrolase localizing to the endomembrane and a chloroplast-localizing ATP binding protein. The contrasting genetic basis of tolerance in the two mutants was taken to imply that CdS QDs tolerance can be achieved by the activation of non-identical master switches. A transcriptomic analysis of wild type (wt) *A. thaliana* plants exposed to Cd^2+^ ions revealed that a completely different gene set was activated, meaning that the pathway leading to CdS QD tolerance must be unrelated to that determining the response to Cd^2+^ stress.

In contrast to a wealth of transcriptomic data sets, the understanding of the proteomic response to ENM exposure is rather limited. In general, the statistical correlation between transcript and protein abundance in eukaryotic cells is poor (Gygi et al., 1999; Hajduch et al., 2010), a phenomenon ascribed largely to the major role played by post-transcriptional modification (Maier et al., 2009). The aim of the current study was to supplement the documented transcriptomic and phenotypic responses of *A. thaliana* to CdS QD exposure (Marmiroli et al., 2014) with a robust set of proteomic data, collected using a double liquid chromatography separation system well-proven to resolve the complex protein mixture present in a plant matrix (Marmiroli et al., 2013).

## Materials and methods

### Plant material

*A. thaliana*, accession *Landsberg erecta* (L. Heyn) mutants atnp01 and atnp02 were isolated by screening of 378 mutant lines obtained from the (Nottingham) European Arabidopsis Stock Centre (uNASC; http://arabidopsis.info/), for resistance to CdS QDs as described by Marmiroli et al. (2014). The same paper, reports also a genetic and molecular characterization of the two mutants.

### Physical properties of the CdS QDs

The CdS QDs utilized during all the experiments had a bulk density of 4.82 g cm^−3^ and a diameter of about 5 nm, they were synthesized following Villani et al. (2012). Cadmium represented ~78% of the dry weight. The CdS QDs were the same batch used into the previous transcriptomic work (Marmiroli et al., 2014).

### Seed germination, growth, and treatments

Twenty-five seeds of *A. thaliana* wild type (wt) and atnp01 and atnp02 were sawn on Petri dishes containing Murashige and Skoog (MS) nutrient medium (Duchefa Biochemie, Haarlem, Netherlands) containing 1% w/v sucrose and solidified with 0.8% w/v agar, then placed in the dark, under controlled conditions in a growth chamber. After germination, seedlings were grown at 24°C, with relative humidity of 30%, and under a 16-h photoperiod (light intensity 120 μM m^−2^ s^−1^ photosynthetic photon flux) in the same MS medium in the absence of CdS QDs for 14 days. Seedlings were transferred to MS medium containing 80 mg L^−1^ CdS QDs (treatment) or 0 mg L^−1^ (control) and grown for a further 21 days, as above. Plantlets were then removed from the medium, carefully washed with distilled H_2_O and used for protein extraction.

### Protein samples preparation

Crude proteins of wt and the two mutant lines in untreated and treated (80 mg L^−1^ CdS QDs) conditions were extracted by using MgSO_4_-gbased buffer. A total amounts of 1 g of frozen plants for wt and mutants and for both treatments were finely ground in liquid nitrogen with a ceramic mortar and pestle, adding SiO_2_ sand (Sigma-Aldrich, St. Louis, MO, USA), to encourage breakage of the cell walls. The fine powder obtained was resuspended in 50 mM Tris [tris (hydroxymethyl) aminomethane] HCl pH 7.8, 10 mM MgSO_4_, 0.1% [v/v] β mercaptoethanol and 0.1% [v/v] Protease Inhibitor Cocktail (Sigma-Aldrich). The crude mixture was sonicated for 10 min at 35 kHz (Transsonic T460, Elma Schmidbauer GmbH, Singen, Germany) and then the solution was placed in ice for 40 min. After 10 min more of sonication, the sample was centrifuged in a precooled rotor spun at 16,000 × g for 5 min at 4°C (Micorfuge 22R Centrifuge, Beckman Coulter, Fullerton, CA, USA). The pellet, containing the larger cellular residues and SiO_2_ sand, was discarded and the supernatant centrifuged at 16.000 × g for 30 min at 4°C. The upper phase was pipetted into other 15 ml tubes and stored at –20°C for further analysis. Three biological replicates were produced for crude protein extracts from wt and mutants.

### Protein quantification

Proteins were quantified using the Quick Start Bradford Protein Assay (BioRad, Hercules, CA, USA); The protein-dye formed was detected at 595 nm with spectrophotometric assay (Uvikon 931, Kontron Instruments) with a standard curve from different dilutions of BSA (Bovine Serum Albumin; Sigma-Aldrich). The BSA dilutions and sample dilutions were prepared in a suitable Chromatofucusing (CF) Start buffer for the next step of two-dimensional liquid chromatography (2D-LC).

### PD10 desalting column

Protein extracts were desalted and equilibrated using PD-10 Desalting Workmade disposable columns (GE-Healthcare Biosciences, Uppsala, Sweden) containing prepacked Sephadex G-25 Medium with exclusion limit of 5000 Da. PD-10 column equilibration was performed by using ~25 ml of CF Start buffer (Eprogen, Downers Grove, IL, USA) and the sample was then eluted with 3.5 ml of CF start buffer. The capacity of the system allows the loading of up to 2.5 ml of sample, with a range of loading capacity between 0.5 and 5 mg of protein per sample.

### Two dimensional liquid chromatography

Three milligram of protein extract were separated by 2D-LC for each sample. Separation was performed with ProteomeLab™ PF2D by Beckman Coulter equipped with: HPCF-1D column 250 × 2.1 mm internal diameter, 300 Å pore size and HPRP C18 column 4.66 mm length × 3.3 mm internal diameter, 1.5 μm particle size (Eprogen). Proteins are separated in the first dimension by high-performance chromatofocusing (HPCF), performed on an HPCF-1D column. With this technique, proteins bound to a strong anion exchanger followed by elution with a continuously decreasing pH (8.5–4.0) gradient. The pH gradient was generated in the column by two buffers: Start Buffer (SB) and Eluent Buffer (EB; Eprogen). The calibration of both buffers was an important step: SB and EB were sonicated for 5 min and then their pH was adjusted to 8.5 and 4.0 respectively using either a saturated solution (50 mg/ml) of iminodiacetic acid (Sigma-Aldrich) if the buffer was too basic, or 1M NH_4_OH (J.T. Baker, Deventer, Holland) if the buffer was too acidic. The column was first equilibrated to the initial pH 8.5 using CF Start buffer at a flow rate of 0.2 ml min^−1^ for 3 h. After this step, 5 ml of sample were injected the column for the first dimension CF. Twenty minutes from sample injection, the first dimension pump switches to the CF Eluent buffer (pH 4) at a flow rate of 0.2 ml min^−1^. The interaction of the column filling with the CF Eluent buffer produced a gradually decreasing pH gradient that traveled through the column as a retained front. The pH gradient affected the proteins net charge and their adsorption/desorption to the positively-charged matrix of the column, causing protein separation in the effluent. The pH of the mobile phase was monitored on-line by a post-detector pH flow cell. The proteins were eluted based on their isoeletric point (pI), measured the absorbance at 280 nm, and collected in a 96 deep-well plate by a fraction collector according to pre-determined pH decrements of 0.4 pH units during the gradient, or in 1 ml volumes when the pH did not change. At the 115th min the most acidic proteins were recovered by washing the column with 1M NaCl 30% n-propanol [v/v] for 15 min. The column was finally washed with water for 45 min; the CF separation took of total of ~185 min.

The eluent from the 1st dimension was injected into the 2nd dimension, a high-performance reversed-phase chromatography (HPRP) based on protein hydrophobicity. HPRP was carried out in a C18 column. The mobile phase consisted of A: 0.1% TFA (Trifluoroacetic Acid; J.T. Baker) in water and B: 0.08% TFA in Acetonitrile (J.T. Baker). Separation was performed at 0.75 ml min^−1^ with an increasing gradient of B. During the first 2 min 100% of solvent A was pumped into the column; in the next 35 min the gradient was created in the column by switching the flow from 0 to 100% solvent B; this is followed by 100% B for 4 min and 100% A for 9 min. In order to obtain a better resolution, the separation was done at 50°C. The eluent from the second dimension was monitored by a second high performance UV/VIS detector at 214 nm, that provided a more universal and sensitive detection of proteins via peptide bonds. Fractions were immediately collected in eppendorf tubes for MS analysis by using an automated fraction collector.

### Protein identification

Matrix-assisted laser desorption/ionization time-of-flight mass spectrometry (MALDI-TOF/MS) analysis was carried out using a 4800 Plus MALDI-TOF/TOF™ (AB SCIEX, Framingham, MA, USA) equipment. Eluted fractions were evaporated to a final individual volume of 10 μl, using a Speed Vac Concentrator 5301 (Eppendorf AG, Barkhausenweg, Hamburg, Germany). Protein digestion was performed by incubating each fraction in 25 mM NH_4_HCO_3_ and 2 mM DTT (DL-Dithiothreitol) in a water bath at 60°C for 1 h. The alkylation of the reduced sulfhydryl groups was carried out adding 1 mM Iodoacetamide, at 25°C, for 30 min in the dark, and then 10 μL of Trypsin (125 μg ml^−1^) in 50 mM NH_4_HCO_3_ were added. Digestion was carried out at 37°C for 24 h. The samples digested were then purified and concentrated with a ZipTipC18 using the procedure recommended by the manufacturer (Millipore Corporation, Billerica, MA, USA). Then 1 μL of each purified peptide was spotted directly onto a stainless steel MALDI target plate with 1 μL of a saturated solution of α-cyano-4-hydroxycinnamic acid in 0.1% TFA:ACN (2:1, v/v). The solution was allowed to dry at room temperature and a spot was produced. Positively charged ions were analyzed in reflectron mode. The spectra were obtained by random scanning of the sample surface with an ablation laser. A mass range of 10,000–100,000 Da was used, and about 400 laser shots were averaged to improve the signal-to-noise ratio. Calibration was performed by a ProteoMass Protein MALDI-MS Calibration kit (Sigma-Aldrich). Two technical replicates for each spectrum were analyzed by MS, and peptides common to all of the resolved spectra were considered for protein identification.

### Statistical and bioinformatics analysis

ProteoVue software (Eprogen) was utilized to convert chromatographic intensities from the 2D-LC of each pH fraction into a band intensity format. This produced a highly detailed map with the dimensions of hydrophobicity and pI. The 2D-LC maps could be viewed in several colored formats where the color intensity was proportional to the relative intensity of each chromatographic peak. DeltaVue software (Eprogen) was utilized for the differential analysis of corresponding fractions from two different sample sets. This software compared chromatogram peaks corresponding to the same protein in the two samples, allowing quantification by a subtractive analysis. A differential map was produced by point-to-point subtraction and it is viewed between the two original sample sets. Mass spectra were analyzed using the mMass software package (http://www.mmass.org/; ver. 5.5, by Martin Strohalm) and the peak list for each mass spectra were obtained. Peptide mass fingerprinting analysis was carried out with the Mascot program (http://www.matrixscience.com). Proteins were identified by searching against Swiss-Prot database of *A. thaliana* (thale cress). The following parameters were used for database search: mass accuracy below 100 ppm, maximum of one missed cleavages by trypsin, carbamidomethylation of cysteine as fixed modifications, oxidation of methionine as variable modifications. The search was based on the monoisotopic masses of the peptides. For mass-spectrometry (MS) analyses, three technical replicates for each spectrum were performed. For proteins identification, only peptides in common to all the resolved spectra were considered.

The gene loci found in the UniProt were searched in TAIR database (https://www.arabidopsis.org/) for the corresponding *A. thaliana* proteins names, description, and GO annotations.

Heat maps of selected proteins were generated by TreeView v1.60 software. Gene Ontology (GO) analysis (Harris et al., 2004) visualized with pie charts were generated by VirtualPlant v1.3 (http://virtualplant.bio.nyu.edu/cgi-bin/vpweb/virtualplant.cgi) applying a p (calculated according to Bonferroni test) cutoff value of 0.05. Venn diagrams were generated by Venny 2.0 (http://bioinfogp.cnb.csic.es/tools/venny/index.html). The correlation between mRNA and protein levels was calculated using the Pearson product moment correlation coefficient in Microsoft Excel 2010.

## Results

### Proteomic data management and visualization

**Figure 1:**
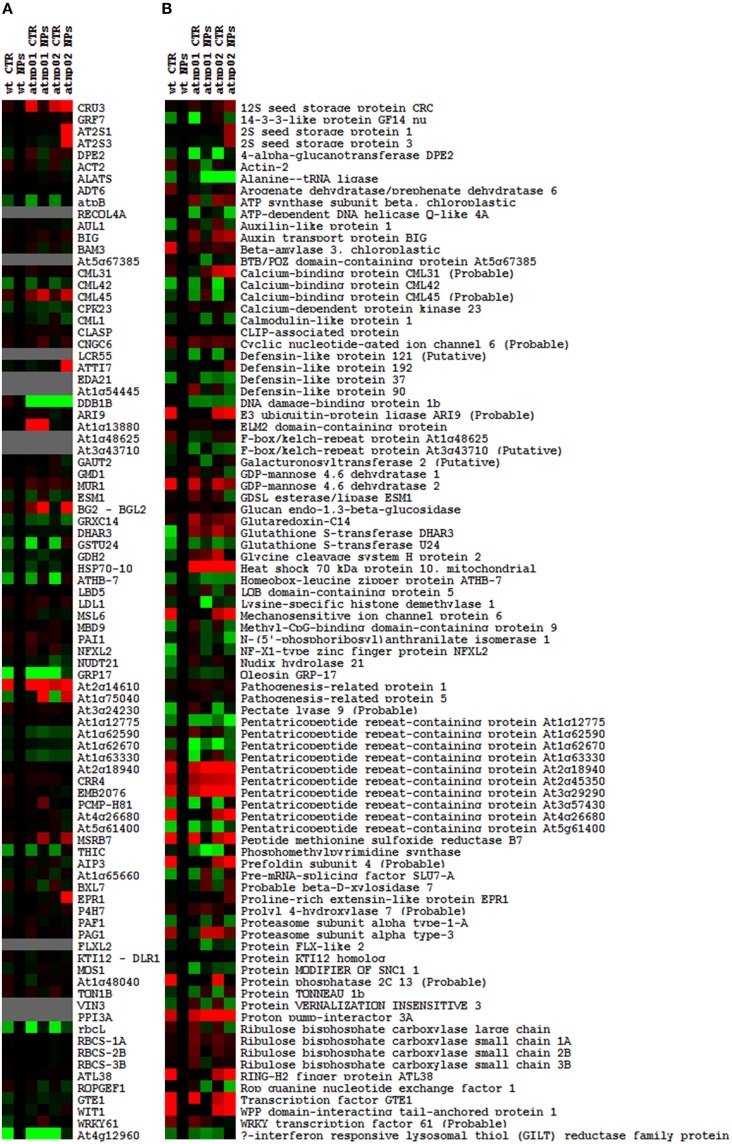
**Heat maps of *A. thaliana* wt and mutant lines atnp01 and atnp02 not treated and treated with 80 mg L^−1^ CdS QDs drawn with TreeView software**. Heat map of the transcriptomic data, the probe “wt treated” was used as calibrator (black column). Up-regulated genes compared to the calibrator are shown in shades of red and down-regulated genes in shades of green **(A)**. Heat map of the proteomic data, “wt treated” was used as calibrator (black column). Proteins more abundant in the sample compared to the calibrator are shown in shades of red, and those less abundant in the sample compared to the calibrator in shades of green **(B)**.

The proteomic separation identified around 600 proteins in the extracts of wt plants and of each of the two mutants exposed and not to CdS QDs. Coupling treated and not treated results for wt, atnp01 and atnp02 three subset of about 1200 proteins were found. The use of DeltaVue software led to the elaboration of a “differential map” for each genetic comparison, where each “band” corresponded to a unique protein and where each virtual band's intensity was proportional to the protein's relative abundance, measured against its abundance in the non-treated control sample. In order to assess which of the intensity ratios were statistically significant, their log_10_'s were grouped into frequency categories, producing a normal distribution; only those proteins associated with a ratio differing from the mean by at least two standard deviations (±) were taken forward for identification, following the strategy outlined by Marmiroli et al. (2013). On this basis almost 200 proteins were selected, but of these, only 130 were abundant enough to be subjected to MALDI-TOF/MS. The identification of some of the proteins using mass fingerprinting was not possible due to low scores, so finally 88 proteins were identified with any statistical confidence. The sets of differentially expressed proteins are listed in Table 1, and a global heat map is presented in Figure 1B: the chosen calibrator was the treated wt plant, because this was found to most clearly highlight the differences between the set of samples, while also allowed direct comparisons to be made with established transcriptomic data (Marmiroli et al., 2014; Figure 1A).

**Table 1:** **atnp01 and atnp02 proteins influenced by mutations and by exposure to 80 mg L^−1^ CdS QDs, separated by 2D-LC and identified by MALDI-TOF/MS and *in silico* analysis**.

**Frac.^a^**	**RT^b^**	**Protein name (UniProt database)^c^**	**Accession no.^d^**	**Gene^e^**	**Locus^f^**	**Probe^g^**	**Mass^h^**	**pI^i^**	**Score^j^**	**Expect^k^**	**Match^l^**	**Cov.^m^ (%)**
14	20.15	12S seed storage protein CRC	CRU3_ARATH	CRU3	At4g28520	253767_AT	58,235	6.99	54	0.047	18	23
27	24.50	14-3-3-like protein GF14 nu	14337_ARATH	GRF7	At3g02520	258489_AT	29,920	4.74	39	1.5	4	18
17	19.32	2S seed storage protein 1	2SS1_ARATH	AT2S1	At4g27140	253904_AT	19,013	5.70	50	0.16	9	31
11	20.00	2S seed storage protein 3	2SS3_ARATH	AT2S3	At4g27160	253895_AT	18,762	7.86	51	0.11	11	38
24	14.91	4-alpha-glucanotransferase DPE2	DPE2_ARATH	DPE2	At2g40840	245094_AT	110,562	5.54	49	0.18	14	14
7	20.18	Actin-2	ACT2_ARATH	ACT2	At3g18780	257749_AT	42,078	5.37	62	0.008	11	37
29	34.09	Alanine–tRNA ligase	SYA_ARATH	ALATS	At1g50200	262468_AT	111,275	6.05	45	0.38	13	21
13	20.43	Arogenate dehydratase/prephenate dehydratase 6	AROD6_ARATH	ADT6	At1g08250	261758_AT	45,059	6.11	41	0.96	5	28
8	24.56	ATP synthase subunit beta, chloroplastic	ATPB_ARATH	atpB	AtCg00480	245014_AT	53,957	5.38	45	0.38	7	19
28	26.67	ATP-dependent DNA helicase Q-like 4A	RQL4A_ARATH	RECQL4A	At1g10930	17661_AT	134,654	6.82	45	0.39	12	14
22	16.14	Auxilin-like protein 1	AUL1_ARATH	AUL1	At1g75310	261117_AT	163,087	4.92	60	0.016	37	22
8	23.62	Auxin transport protein BIG	BIG_ARATH	BIG	At3g02260	259128_AT	574,541	5.65	54	0.047	30	7
15	19.30	Beta-amylase 3, chloroplastic	BAM3_ARATH	BAM3	At4g17090	245346_AT	61,713	6.59	40	1.2	20	41
8	26.25	BTB/POZ domain-containing protein At5g67385	Y5738_ARATH	At5g67385	At5g67385	–	68,086	8.16	34	36	3	4
27	18.30	Calcium-binding protein CML31 (probable)	CML31_ARATH	CML31	At2g36180	263903_AT	16,375	4.21	47	0.25	7	53
14	13.37	Calcium-binding protein CML42	CML42_ARATH	CML42	At4g20780	254487_AT	21,191	4.59	55	0.043	7	34
21	17.08	Calcium-binding protein CML45 (Probable)	CML45_ARATH	CML45	At5g39670	249417_AT	24,104	4.93	33	6.9	5	34
4	17.01	Calcium-dependent protein kinase 23	CDPKN_ARATH	CPK23	At4g04740	255306_AT	59,073	6.09	60	0.001	11	23
22	14.95	Calmodulin-like protein 1	CML1_ARATH	CML1	At2g15680	265494_AT	16,3087	4.92	46	0.34	31	18
29	34.09	CLIP-associated protein	CLASP_ARATH	CLASP	At2g20190	265315_AT	160,107	6.72	46	0.34	31	22
2	22.23	Cyclic nucleotide-gated ion channel 6 (Probable)	CNGC6_ARATH	CNGC6	At2g23980	266520_AT	86,299	9.38	45	0.42	12	19
24	15.87	Defensin-like protein 121 (Putative)	DF121_ARATH	LCR55	At3g20997	–	8969	7.51	42	0.85	5	67
14	19.90	Defensin-like protein 192	DF192_ARATH	ATTI7	At1g47540	262431_AT	11,063	6.34	44	0.45	8	55
28	21.11	Defensin-like protein 37	DEF37_ARATH	EDA21	At4g13235	–	9708	9.3	40	1.2	3	36
2	24.91	Defensin-like protein 90	DEF90_ARATH	At1g54445	At1g54445	–	9175	8.44	40	1.4	3	57
18	15.50	DNA damage-binding protein 1b	DDB1B_ARATH	DDB1B	At4g21100	254452_AT	121,178	4.97	44	0.45	6	44
26	23.93	E3 ubiquitin-protein ligase ARI9 (probable)	ARI9_ARATH	ARI9	At2g31770	263463_AT	64,529	4.96	45	0.44	11	19
24	17.90	ELM2 domain-containing protein	D7KQK8_ARAL	At1g13880	At1g13880	259423_AT	48,428	4.18	50	0.15	14	40
14	21.12	F-box/kelch-repeat protein At1g48625	FBK20_ARATH	At1g48625	At1g48625	–	48,704	8.9	51	0.11	11	32
29	34.12	F-box/kelch-repeat protein At3g43710 (putative)	FBK72_ARATH	At3g43710	At3g43710	–	44,064	7.91	40	1.3	8	15
5	23.07	Galacturonosyltransferase 2 (putative)	GAUT2_ARATH	GAUT2	At2g46480	265477_AT	62,393	6.98	34	5.2	4	16
3	25.23	GDP-mannose 4,6 dehydratase 1	GMD1_ARATH	GMD1	At5g66280	247094_AT	40,939	6.61	41	0.94	6	26
23	20.37	GDP-mannose 4,6 dehydratase 2	GMD2_ARATH	MUR1	At3g51160	252121_AT	40,939	6.61	53	0.07	5	22
22	15.87	GDSL esterase/lipase ESM1	ESM1_ARATH	ESM1	At3g14210	257008_AT	44,374	7.59	44	0.49	6	18
19	16.55	Glucan endo-1,3-beta-glucosidase	E13A_ARATH	BGL2	At3g57260	251625_AT	37,338	4.60	44	0.60	9	25
10	23.05	Glutaredoxin-C14	GRC14_ARATH	GRXC14	At3g62960	251197_AT	11,561	7.66	44	0.45	4	66
12	17.40	Glutathione S-transferase DHAR3	DHAR3_ARATH	DHAR3	At5g16710	246454_AT	28,724	7.59	35	4.3	12	41
14	18.51	Glutathione S-transferase U24	GSTUO_ARATH	GSTU24	At1g17170	262518_AT	25,461	5.85	56	0.031	17	47
22	14.51	Glycine cleavage system H protein 2	GCSH2_ARATH	GDH2	At2g35120	266517_AT	17,203	4.97	49	0.18	8	31
27	22.01	Heat shock 70 kDa protein 10, mitochondrial	HSP7J_ARATH	HSP70-10	At5g09590	250502_AT	73,174	3.5	36	3.5	5	9
28	27.65	Homeobox-leucine zipper protein ATHB-7	ATHB7_ARATH	ATHB-7	At2g46680	266327_AT	30,663	5.52	42	0.9	12	50
24	18.36	LOB domain-containing protein 5	LBD5_ARATH	LBD5	At1g36000	260187_AT	14,477	6.73	34	4.9	8	44
19	19.62	Lysine-specific histone demethylase 1	LDL1_ARATH	LDL1	At1g62830	262668_AT	93,767	4.84	64	0.005	10	17
26	24.89	Mechanosensitive ion channel protein 6	MSL6_ARATH	MSL6	At1g78610	263127_AT	97,057	8.73	42	0.8	13	16
22	15.98	Methyl-CpG-binding domain-containing protein 9	MBD9_ARATH	MBD9	At3g01460	258950_AT	243,929	5.34	54	0.059	32	17
2	24.12	N-(5'-phosphoribosyl)anthranilate isomerase 1	PAI1_ARATH	PAI1	At1g07780	259770_S_AT	29,847	8.63	35	3.7	5	32
28	24.68	NF-X1-type zinc finger protein NFXL2	NFXL2_ARATH	NFXL2	At5g05660	250767_AT	105,547	8.63	43	0.67	14	18
27	24.20	Nudix hydrolase 21	NUD21_ARATH	NUDT21	At1g73540	245777_AT	22,793	8.39	52	0.084	4	43
2	21.30	Oleosin GRP-17	GRP17_ARATH	GRP17	At5g07530	250637_AT	53,330	10.34	36	3.5	15	36
4	18.20	Pathogenesis-related protein 1	PR1_ARATH	At2g14610	At2g14610	266385_AT	17,676	8.96	48	0.20	8	35
18	17.80	Pathogenesis-related protein 5	PR5_ARATH	At1g75040	At1g75040	259925_AT	25,252	4.54	53	0.040	16	43
27	23.98	Pectate lyase 9 (probable)	PLY9_ARATH	At3g24230	At3g24230	257243_AT	50,638	8.65	48	0.22	11	31
28	19.63	Pentatricopeptide repeat-containing protein At1g12775	PPR39_ARATH	At1g12775	At1g12775	261194_AT	73,474	6.15	44	0.46	10	28
2	24.33	Pentatricopeptide repeat-containing protein At1g62590	PPR90_ARATH	At1g62590	At1g62590	265106_S_AT	71,899	7.97	53	0.061	6	9
24	15.36	Pentatricopeptide repeat-containing protein At1g62670	PPR91_ARATH	At1g62670	At1g62670	260792_AT	110,562	5.54	50	0.15	10	10
27	19.43	Pentatricopeptide repeat-containing protein At1g63330	PP101_ARATH	At1g63330	At1g63330	265106_S_AT	63,778	6.83	36	2.9	6	10
27	21.94	Pentatricopeptide repeat-containing protein At2g18940	PP163_ARATH	At2g18940	At2g18940	266951_AT	93,433	8.36	36	3.6	13	20
14	24.38	Pentatricopeptide repeat-containing protein At2g45350	PP202_ARATH	CRR4	At2g45350	245129_AT	70,052	6.78	46	0.37	5	10
27	21.44	Pentatricopeptide repeat-containing protein At3g29290	PP262_ARATH	EMB2076	At3g29290	256613_AT	63,160	9.01	38	2.1	5	12
24	27.45	Pentatricopeptide repeat-containing protein At3g57430	PP285_ARATH	PCMP-H81	At3g57430	251631_AT	100,466	6.94	37	2.7	15	15
26	25.63	Pentatricopeptide repeat-containing protein At4g26680	PP338_ARATH	At4g26680	At4g26680	253979_AT	60,010	9.45	47	0.28	12	28
24	13.03	Pentatricopeptide repeat-containing protein At5g61400	PP440_ARATH	At5g61400	At5g61400	247548_AT	75,760	7.91	37	2.5	10	14
26	26.77	Peptide methionine sulfoxide reductase B7	MSRB7_ARATH	MSRB7	At4g21830	254385_S_AT	15,789	8.54	51	0.11	4	36
4	14.49	Phosphomethylpyrimidine synthase	THIC_ARATH	THIC	At2g29630	266673_AT	72,568	6.00	46	0.31	9	22
26	26.02	Prefoldin subunit 4 (probable)	PFD4_ARATH	AIP3	At1g08780	264778_AT	14,985	4.55	41	1	5	54
14	13.68	Pre-mRNA-splicing factor SLU7-A	SLU7A_ARATH	At1g65660	At1g65660	264633_AT	62,281	5.73	41	1.1	12	21
4	11.96	Probable beta-D-xylosidase 7	BXL7_ARATH	BXL7	At1g78060	262181_AT	84,751	8.3	66	0.003	15	22
1	12.50	Proline-rich extensin-like protein EPR1	EPR1_ARATH	EPR1	At2g27380	265644_AT	81,946	10.61	60	0.009	12	19
29	28.05	Prolyl 4-hydroxylase 7 (probable)	P4H7_ARATH	P4H7	At3g28480	257844_AT	36,117	6.32	43	0.64	7	20
16	18.93	Proteasome subunit alpha type-1-A	PSA1A_ARATH	PAF1	At5g42790	249161_AT	30,685	4.99	45	0.4	7	27
29	34.48	Proteasome subunit alpha type-3	PSA3_ARATH	PAG1	At2g27020	266312_AT	27,645	5.93	62	0.008	9	24
19	21.88	Protein FLX-like 2	FLXL2_ARATH	FLXL2	At1g67170	–	39,831	6.93	48	0.21	7	23
15	19.32	Protein KTI12 homolog	KTI12_ARATH	DLR1	At1g13870	259450_AT	34,063	5.97	44	0.73	9	33
29	27.96	Protein MODIFIER OF SNC1 1	MOS1_ARATH	MOS1	At4g24680	254143_AT	153,766	9.39	53	0.067	21	19
7	20.45	Protein phosphatase 2C 13 (probable)	P2C13_ARATH	At1g48040	At1g48040	260722_AT	42,649	4.85	52	0.088	10	25
24	19.84	Protein TONNEAU 1b	TON1B_ARATH	TON1B	At3g55005	251816_AT	29,338	5.06	37	3	14	46
22	17.02	Protein VERNALIZATION INSENSITIVE 3	VIN3_ARATH	VIN3	At5g57380	–	71,128	5.88	39	1.6	16	26
27	31.71	Proton pump-interactor 3A	PPI3A_ARATH	PPI3A	At5g36780	–	66,923	6.07	33	6.2	7	14
2	24.88	Ribulose bisphosphate carboxylase large chain	RBL_ARATH	rbcL	AtCg00490	245015_AT	53,435	5.88	49	0.16	10	23
2	22.76	Ribulose bisphosphate carboxylase small chain 1A	RBS1A_ARATH	RBCS-1A	At1g67090	264474_S_AT	20,488	7.59	101	1.6e-07	12	68
3	22.72	Ribulose bisphosphate carboxylase small chain 2B	RBS2B_ARATH	RBCS-2B	At5g38420	264474_S_AT	20,622	7.59	101	0.00001	13	59
3	23.95	Ribulose bisphosphate carboxylase small chain 3B	RBS3B_ARATH	RBCS-3B	At5g38410	264474_S_AT	20,556	8.22	54	0.048	8	53
26	25.17	RING-H2 finger protein ATL38	ATL38_ARATH	ATL38	At2g34990	267417_AT	34,890	6.71	43	0.64	8	36
3	24.77	Rop guanine nucleotide exchange factor 1	ROGF1_ARATH	ROPGEF1	At4g38430	252975_S_AT	61,380	5.44	40	1.3	8	20
26	26.52	Transcription factor GTE1	GTE1_ARATH	GTE1	At2g34900	257352_AT	43,586	6.04	45	0.37	7	26
26	25.09	WPP domain-interacting tail-anchored protein 1	WIT1_ARATH	WIT1	At5g11390	250363_AT	79,228	4.7	39	1.6	12	13
12	21.17	WRKY transcription factor 61 (probable)	WRK61_ARATH	WRKY61	At1g18860	261429_AT	53,224	6.48	35	4.1	10	18
12	17.80	γ-interferon responsive lysosomal thiol (GILT) reductase family protein	F4JRI7_ARATH	At4g12960	At4g12960	254777_AT	27,196	7.05	38	3.7	14	44

a fraction,

b progressive peak number as given by 2D-LC,

c putative protein identification,

d accession number for the closest match in the UniProt database,

e gene,

f locus,

g probe,

h predicted mass value,

i pI of the closest match in the database,

j score,

K expected value of the database research,

l matches,

m *percentage of coverage of the matching peptide sequence tags derived by applying MASCOT algorithm*.

**Figure 2:**
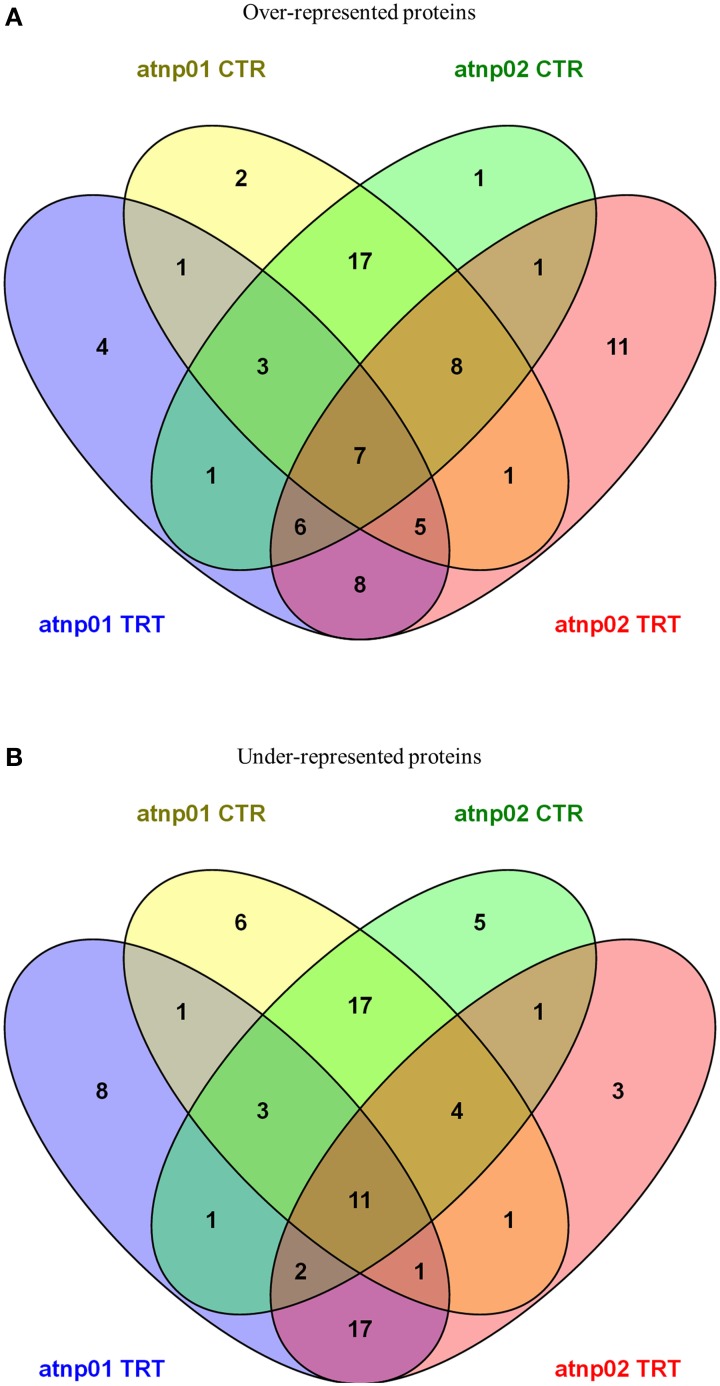
**Venn diagrams for over-represented proteins in atnp01 and atnp02 mutant lines both for control and treatment conditions (A) and under-represented proteins in atnp01 and atnp02 mutant lines both for control and treatment conditions (B)**. Within each subset a natural figure (n ∈ N) denotes the number of members (proteins) included in the subset.

Venn diagrams featuring the differentially represented, both over- and under-represented, proteins in both mutants compared to the wt in both the treated and untreated situation are presented in Figure 2. There were 35 over-represented proteins in the treated atnp01 mutant, and 47 in the atnp02 mutant; of these, 26 were in common between the two comparisons. The respective frequencies of under-represented proteins were 44 in atnp01, 40 in atnp02, and 31 common to both mutants. In the comparison between treated wt and atnp01 plants, nine proteins having the same abundance.

In the comparisons involving non-treated plants, there were 44 over-represented proteins in each mutant, of which 35 were in common. With respect to the set of under-represented proteins: 44 for atnp01, 40 for atnp02, of which 35 in common. Inspection of the data revealed that seven of the over-represented and 11 of the under-represented proteins did not change in abundance either as a consequence of the treatment or as a result of a genetic difference, 17 over- and 17 under-represented were ascribable to genetic differences and eight over- and 17 under-represented ones to the CdS QDs exposure. The atnp01 mutation affected eight proteins (two over-, six under-represented), while the atnp02 mutation affected six proteins (one over-, five under-represented). The CdS QDs treatment altered the expression level of 12 proteins in atnp01 (four over-, eight under-represented) and 14 in atnp02 (11 over-, three under-represented).

### Functional analysis of differentially expressed proteins

A GO analysis was conducted to assign functionality to the set of differentially expressed proteins (Supplementary Figures S1–S4). The most frequently encountered GO class was biological process, followed by molecular function and cellular components. For both the mutants, the over- and the under-represented proteins were classified within the biological process category as involving a cellular process, a metabolism or a response to stimuli.

The over-represented proteins in atnp01 concerned metabolic and cellular processes, response to stimuli and regulation (Supplementary Figure S1), the cellular components interested being extracellular parts, cell parts and organelles. The molecular function of relevance were catalytic, binding but also electron carriers and antioxidants activity. The over- represented proteins in atnp02 concerned metabolic and cellular processes and as for atnp01 response to stimuli (Supplementary Figure S2). Also for the cellular components and molecular functions the similarities were remarkable (Supplementary Figure S2).

The proteomic response of the two mutants evidenced, in the condition of treatment for atnp01, under-represented proteins in the biological process metabolisms, cellular response to stimuli, cellular components organization with at this level a predominance of cell part and organelle (Supplementary Figure S3). The molecular function involved were: catalytic activity, binding and transport (Supplementary Figure S3). In the mutant atnp02 the under-represented concerned proteins of the cell metabolism and developmental process, but also cellular components organization and response to stimuli (Supplementary Figure S4). Cell part and organelles were the more affected with molecular function in the class of catalytic activity and binding as majority.

### Comparison between the transcriptome and the proteome

**Figure 3:**
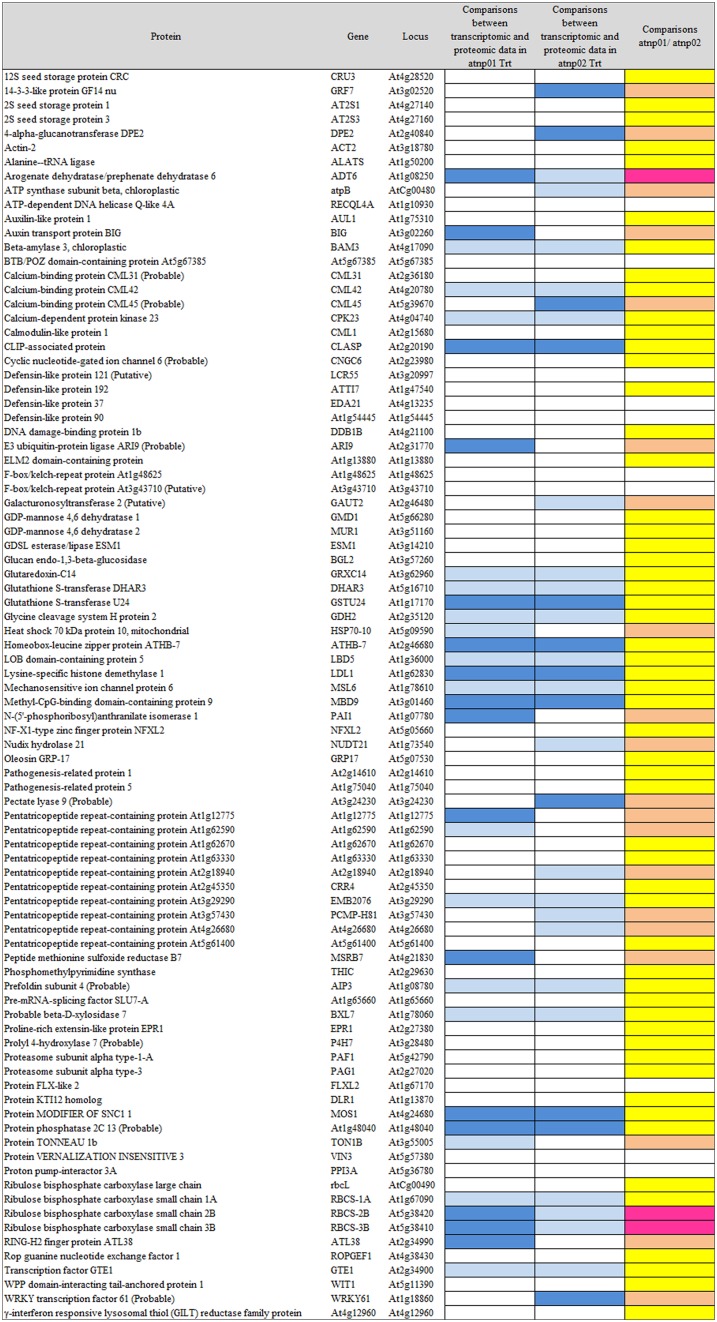
**Visualization as “heat map” of the comparison between transcriptomic data and proteomic data**. In column 4 and 5, white rectangles indicate concurrency between transcript level and protein abundance, light blue indicates that high level of transcript has a low protein abundance, blue indicates that low level of transcript has high protein abundance. In the last column, yellow rectangles are for consistent behavior between the two mutants in the transcriptomic-proteomic cascade, orange is for contrasting behavior, specifically, pink is for a markedly opposite trend.

Based on the transcriptome description provided by Marmiroli et al. (2014), 78 of the 88 proteins were assignable an encoding transcript (Figure 1). The Pearson product moment correlation coefficients (r) for transcript and protein representation for the two mutants were, respectively, 0.126 and 0.197. Figure 3 shows a comparison between protein and transcript over-represented with respect to gene product, but under-represented with respect to transcript (column 4 and 5). In atnp01, 46 of the proteins (59%) exhibited a matching level of transcript and protein (“concurrent” gene products), while 16 were over-represented even though their RNA was underexpressed, and 16 behaved in the opposite manner; these 32 gene products were termed “non-concurrent.” In atnp02, there were 44 (57%) concurrent and 34 non-concurrent proteins, of which 12 (15%) were over-represented with respect to gene product, but under-represented with respect to transcript, and 22 (28%) *vice versa*. In atnp02, 57% were concurrent, while of the non-concurrent ones, 15%, the reverse holds for the 28% of the proteins (Table 2). Since the studied protein set was so much smaller than the number of relevant transcripts, reported in Marmiroli et al. (2014) (88 vs. 456), a correlation analysis based on either Pearson's P or Kendall's τ was considered to be unsuitable. In order to recognize an association between transcript and protein abundance induced by the Cd QDs, the behavior of the two mutants was compared: either the direction of change of the transcript abundance matched that of the protein in both mutants, or it did not. Thus, two broad groups were defined comprising a constant, invariable, or a variable, at times unquestionably opposite, behavior, which describes the trend of protein production rate against the backdrop of transcript. The final column in Figure 3 depicts the general cascade from transcript to gene product induced by the treatment in the two mutants. In all, for 71% of the gene products, protein representation reflected the behavior of the matching transcript, while for the remaining 29%, there was no apparent relationship; in 3% of the cases, a particularly high transcript expression was matched by a particularly low level of protein representation or *vice versa* (Table 2).

**Table 2:** **Comparison between transcriptomic and proteomic data**.

		**atnp01 (%)**	**atnp02 (%)**	**Comparisons atnp01/atnp02 (%)**
% Concurrent		58.97	56.41	70.51
% Non-concurrent	High transcript low protein	20.51	15.38	29.49
	Low transcript high protein	20.51	28.21	

### Identification of specific proteins

In Supplementary Tables S1, S2 is reported all the bibliography relevant to each protein mentioned in this sub-heading.

The proteome of both mutants differed from that of the wt, both when the plants were growth under control conditions and when they were exposed to CdS QDs. In the absence of the stress treatment, just two proteins were specifically over-represented in atnp01 and one in atnp02 (Figure 2A). One of the former was an alanine-tRNA ligase expressed in the mitochondria and the chloroplasts, which forms part of the response to both salinity and Cd^2+^ stress; the other was N-(5′-phosphoribosyl) anthranilate isomerase 1, an enzyme which catalyzes a step in the tryptophan synthesis pathway, and is active in guard cell chloroplasts. The sole atnp02-specific over-represented protein of unknown function was a member of the pentatricopeptide repeat superfamily active in the mitochondria (Supplementary Table S1). There were six atnp01-specific under-represented proteins in the non-stressed plants (Supplementary Table S2). These comprised (1) a calcium-binding protein CML31 localizing to the nucleus, (2,3) two pentatricopeptide repeat-containing proteins of unknown function expressed in the mitochondria, (4) a pentatricopeptide repeat-containing protein member of the PCMP-E subfamily involved in RNA editing in the chloroplast, (5) an alpha type 3 proteasome subunit active in both the cytosol and various organelles, and involved in glycolysis, photorespiration, proteolysis, the hyperosmotic response, the response to various abiotic stress agents (including Cd^2+^) and water transport, and (6) a proton pump-interactor 3A, which may be responsible for the regulation of plasma membrane ATPase activity and proton transport. There were five under-represented proteins specific to atnp02 (Supplementary Table S2). These comprised (1) a calcium-binding protein CML42 involved in protein binding and trichome branching, (2) a glucan endo-1,3-beta-glucosidase which participates in a MAPK cascade and in a variety of other processes, and localizes within the apoplast, cell wall, chloroplast and vacuole, (3) a homeobox-leucine zipper protein ATHB-7 thought to be a transcription factor acting in a signal transduction pathway mediating the drought response, and (4,5) the two pathogenisis-related proteins PR1 and PR5, present in the apoplast, cell wall and extracellular regions.

Exposure to CdS QDs resulted in the specific over-representation of four proteins in atnp01 and 11 in atnp02 (Figure 2A; Supplementary Table S1). The former set comprised (1) a calcium-binding protein CML45 of unknown function, (2) a putative defensin expressed extracellularly, (3) a KTI12 homolog expressed in both the cytoplasm and the nucleus, and involved in 5-carbamoylmethyluridine metabolism and also in the regulation of transcription and in tRNA modification and, (4) a WRKY transcription factor involved in the response to Zn^2+^. The 11 atnp02-specfic proteins were as follows: (1) a plastid-localized arogenate dehydratase involved in phenylalanine synthesis, in anthocyanin accumulation in response to UV irradiation and in the vernalization response, (2) a probable E3 ubiquitin-protein ligase, (3) an F-box/kelch-repeat protein of unknown function, (4) a mechanosensitive ion channel protein responsible for ion transmembrane transport, (5) a mitochondrion localizing member of the tetratricopeptide repeat-like superfamily, (6) a mitochondrion localizing methionine sulfoxide reductase involved in oxidation-reduction, protein repair and the response to singlet oxygen, (7) a plastid localizing phosphomethylpyrimidine synthase involved in pathogen detection, glucosinolate and maltose metabolism and several other processes, (8) a prefolding subunit 4, expressed in the cytosol and the nucleus, (9) a membrane RING-H2 finger protein associated with Zn^2+^ binding, (10) a nuclear GTE1 transcription factor involved in the regulation of germination, and (11) a nuclear WPP domain-interacting tail-anchored protein 1 involved in lateral root development and nucleocytoplasmic transport. Overall, a somewhat larger number of proteins was over-represented in atnp02 than in atnp01.

Exposure to CdS QDs resulted in the specific under-representation of eight proteins in atnp01 and three in atnp02 (Figure 2B; Supplementary Table S2). The former set comprised: (1) an extracellular 2S seed storage and lipid binding protein, (2) a chloroplastic ATP synthase subunit β, involved in photosynthesis and aspects of the biotic and abiotic stress response, (3) BIG, a cytosolic protein, involved in auxin polar transport, auxin-activated signaling, inflorescence morphogenesis, lateral root formation and development, and the anti-fungal response, (4) a putative galacturonosyltransferase 2, responsible for carbohydrate and pectin synthesis and cell wall organization, (5) a chloroplast nudix hydrolase 21, (6) a structural constituent of the extensin-like EPR1 involved in cell wall modification, seed lipid storage, embryo development, dormancy and germination, and sugar-mediated signaling, and (7,8) two RuBisCO small subunits (RBCS2B and RBCS3B). The three specifically under-represented atnp02 proteins were: (1) esterase/lipase ESM1 involved in photosynthesis, starch synthesis, pest/pathogen defense, (2) an extracellular pectate lyase, and (3) a mitochondrion localizing pentatricopeptide repeat-containing protein acting as an adenylate cyclase.

Among the proteins showing altered representation in both mutants (either in the control plants and/or in the CdS QDs exposed ones), seven were over-represented and 11 under-represented (Figures 2A,B; Supplementary Tables S1, S2). The former group comprised: (1) a 12S CRC protein responsive to abscisic acid and associated with lipid storage, protein ubiquitination, germination, seed maturation and sugar-mediated signaling, (2) a calcium-dependent protein kinase 23 involved in abscisic acid-activated signaling, intracellular signal transduction, protein phosphorylation and the response to Cd^2^, (3) glutaredoxin C14, (4) DHAR3—a chloroplast-localizing dehydroacorbate reductase involved in protein glutathionylation and toxin catabolism, (5) a mitochondrion localizing glycine cleavage system H protein 2, (6) the nuclear pre-mRNA splicing factor SLU7-A, and (7) the RuBisCO small subunit RBCS1A. The 11 down-regulated proteins were: (1) a chloroplast and cytosolic 4- alpha glucanotransferase DPE2, involved in the sensing of the circadian rhythm, polysaccharide and starch metabolism and cell wall organization, (2) actin2, a cytosolic protein involved in anthocyanin accumulation, cellulose metabolism, the response to various abiotic stresses and water transport, (3) a nuclear ATP-dependent DNA helicase Q-like 4A involved in DNA recombination, repair and replication, and the cellular responses to DNA damage and low temperature stress, (4) a BTP/POZ domain-containing plasma membrane protein responsible for protein ubiquitination, (5) a CLIP-associated protein involved in anthocyanin accumulation, cellulose metabolism, polysaccharide and cell wall synthesis and root hair elongation, (6) a nuclear DNA damage binding protein 1b involved in DNA repair, cell division and embryo and reproductive structure development, (7) a nuclear F-box/kelch-repeat protein belonging to the galactose oxidase/kelch repeat superfamily, (8) a pentatricopeptide repeat-containing protein required for the 5′ processing of nad9 and cox3 mRNAs in the mitochondria, (9,10) two mitochondrial pentatricopeptide repeat-containing proteins of unknown function, and (11) an SNC1 modifier involved in the regulation of gene expression, glucuronoxylan metabolism, and nuclear-transcribed mRNA catabolism.

Exposure to CdS QDs resulted in an increase in the number of over and under-represented proteins in both mutants (Figures 2A,B; Supplementary Tables S1, S2). The over-represented proteins were eight: (1) a chloroplast-localized beta-amylase 3 involved in maltose and starch synthesis and the response to low temperature, (2) a cyclic nucleotide-gated ion channel 6, (3) a GDP-mannose 4,6-dehydratase 2, involved in *de novo* GDP-L-fucose synthesis and GDP-mannose and glucose metabolism, (4) a nuclear LOB domain-containing protein 5 of unknown function, (5) a chloroplast pentatricopeptide repeat-containing protein involved in chloroplast RNA and mRNA processing, (6) a probable beta-D-xylosidase 7 involved in carbohydrate metabolism, (7) a probable prolyl 4-hydroxylase 7, involved in oxidation-reduction, (8) the cytoskeletal protein TONNEAU 1-B, which is probably involved in cortical cytoskeleton organization and microtubule organization. The set of under-represented proteins in both mutants comprised 17 proteins: (1) a chloroplast auxilin-like protein 1, which binds to certain heat shock proteins and is associated with protein folding, (2) a calmodulin-like protein 1 localizing to the mitochondria and the plasma membrane, (3,4) two extracellular defensin-like proteins involved in anti-fungal defense, embryo sac development and transition metal ion transport, (5) a GDP-mannose 4,6-dehydratase 1, (6) a glutathione S-transferase U 24 involved in fatty acid beta-oxidation as well as in protein and toxin catabolism, (7) a 70 kDa heat shock protein 10 involved in protein folding, peroxide neutralization and the response to various abiotic stresses (including Cd^2+^), (8) a nuclear lysine-specific histone demethylase 1 involved in histone H3-K4 methylation, histone deacetylation, oxidation-reduction and the regulation of transcription, (9) a methyl-CpG-binding domain-containing protein 9 involved in cell wall organization, the regulation of transcription, embryo development, the sensing of photoperiod, flowering and secondary shoot formation, (10) a nuclear NF-X1-type zinc finger protein NFXL2 involved in sensing the circadian rhythm, floral development and the regulation of transcription, (11) oleosin GRP-17, a lipid-binding protein involved in lipid storage, cell wall modification and pollen development, (12) a proteasome subunit alpha type 1-A endowed with endopeptidase and peptidase activity and involved in fatty acid oxidation, protein catabolism and the response to As stress, (13) an FLX-like 2 protein of unknown function expressed in the guard cells, (14) the nuclear protein vernalization insensitive 3, which forms part of the low temperature-induced regulation of gene expression, (15) a RuBisCO large subunit, (16) the ROP guanine nucleotide exchange factor 1 involved in anthocyanin accumulation in response to UV irradiation, polysaccharide synthesis, the regulation of hormone levels and pollen tube growth, root hair elongation and root morphogenesis and (17) an extracellular gamma-interferon responsive lysosomal thiol (GILT) reductase with catalytic activity.

## Discussion

The most frequently reported toxicity problem associated with ENMs is oxidative stress (Pujalté et al., 2011; Ma et al., 2015). When taken up, they can drive down the cellular content of antioxidants and/or increase its production of reactive oxygen species (ROS; Maysinger and Lovric, 2007; Mahmoudi et al., 2011; Santos et al., 2012). A better understanding of the properties of these materials, along with technical improvements in their synthesis, should provide the means to reduce their hazard: examples are the use of biocompatible coating materials and the exploitation of surface functionalization, which both help mask the particles' surface reactivity (Lynch et al., 2014; Burello and Worth, 2015). The toxicity of CdS QDs has been related to not just their small size but also their high surface charge and reactivity, photolytic activity, shape, composition, and mechanical stability (Favero et al., 2006; Maysinger and Lovric, 2007). Toxicity tests based on conventional pharmacokinetic and/or pharmacodynamic approaches (Holford, 2007; Steele and Austin, 2009) may be inadequate to identify the full range of potential hazards posed by CdS QDs. This realization explains the present application of a genotoxicological approach.

Transposon mutagenesis has succeeded in identifying two *A. thaliana* mutants (atnp01 and atnp02) able to tolerate a level of CdS QDs sufficient to strongly compromise the growth of a wt plant (Marmiroli et al., 2014). Comparing the transcriptomes of these mutants with that of the wt has provided a ready means to define which genes which were up- or down-regulated in one or both of the mutant(s), both in non-stressed conditions and when the plants were exposed to CdS QDs (Marmiroli et al., 2014). Here, the comparisons have been extended to the protein level, by exploiting platforms able to identify not just specific gene products but also some of their post-translationally modified forms. Combining these data with those acquired from other omics platforms is the aspiration of current system biology strategies, which aim to define the complex pathways and networks involved in response to different external stimuli (Chen and Harmon, 2006; Jorrín-Novo et al., 2015; Wang et al., 2015). Of particular note are the two proteins DRL and ELM, the encoding transcripts for which were both abundant in atnp01 plants whether or not the Cd QD treatment was imposed; despite this, both proteins were only slightly over-represented (Figure 1) confirming their epistatic role (Marmiroli et al., 2014). The two mutants differed quite markedly at the proteomic level: while atnp01 has a mixed change in its proteins abundance, ready to cope with general stress situations, the mutations affecting atnp02 were more closely related to the response to oxidative stress. Many of the proteins altered in their level of expression in atnp01 were concerned with DNA transcription, lipid binding and the auxin response; in contrast, in atnp02, although there was also an effect on some proteins involved in DNA transcription, a range of other functions were also modified, including protein metabolism, cell wall formation and photosynthesis. Note that the oxidative stress response is triggered by excessive amounts of ROS, which not only induces changes in DNA transcription, but also triggers the metabolism of proteins, starch and sugars (Desikan et al., 2001; Mittler et al., 2004; Fujita et al., 2006; Foyer and Noctor, 2011). In both mutants, there was an over-representation of proteins associated with the oxidative stress response and an under-representation of those associated with DNA and RNA processing and with cell development.

An over-representation of lytic proteins and an under-representation of stress-related and hormone-regulated proteins was an unexpected feature of the CdS QD treatment. Characteristic of an oxidative stress response was the up-regulation of sugar metabolism, a disturbance in phytohormone levels and the prominence of glutathione/ascorbate cycle related enzymes (Couée et al., 2006; Foyer and Noctor, 2011; Villiers et al., 2011). There was overall little commonality between the two mutants with respect to either which proteins were over- or which were under-represented, compared with the WT (Supplementary Figure S5). This pointed to possibly divergent phenotypic traits as a result of the over-represented proteins in respect to possibly convergent traits as a result of the under-represented proteins in atnp01 and atnp02. Nevertheless, the numbers of altered proteins expressed in the two mutants in plants not exposed to CdS QDs were rather similar to one another, even though the proteins differed so widely in type, function and cell localization (Supplementary Figure S5).

A growing body of literature has confirmed that transcription levels in eukaryotes are poorly correlated with the levels of their encoded products (Griffin et al., 2002; Lan et al., 2012). This uncoupling is assumed to reflect the action of a number of cellular phenomena, notably the influence of RNA secondary structure, the activity of regulatory proteins and regulatory siRNAs, codon bias and codon adaptation, ribosomal density, and protein half-life (Gygi et al., 1999; Hajduch et al., 2010). The Pearson r correlation coefficients for atnp01 and atnp02 were, respectively, 0.126 and 0.197, levels which confirm the anticipated poor correlation between transcriptome and proteome. As an alternative means of linking the two data sets, a qualitative rather than a quantitative view was taken of the relationship between each transcript/protein pair (Figure 3). The criterion adopted highlighted the direction rather than the extent to which the amount of a particular couple of cognate transcript and protein was affected. The number of up- and down-regulated genes which, in this sense, matched the behavior of their encoded protein was quite similar in the two mutants (46 and 44), of which 31 were represented in both mutants (Supplementary Figure S6). There were 16 genes in atnp01 associated with an increased expression of transcript but a decreased representation of protein, and 12 behaving in this manner in atnp02. The frequency of genes responding in the opposite direction (low transcript/high protein abundance) was 16 in atnp01 and 22 in atnp02. Overall, therefore, about 59% of differentially represented proteins in atnp01 and 56% of those in atnp02 behaved in a concurrent manner (Table 2), a frequency which is quite consistent with the outcome of cognate studies in other eukaryotes (Hajduch et al., 2010). It is thus possible to argue that the mutants' responses were split into two almost equally-sized parts: one was a shared response, and the other was specific to the mutant. For 70% of the reprogrammed genes, at a certain level of transcript corresponded the same amount of protein, either over or under-represented in the two mutants. On the other hand, without referring to the nature of the type of change within a single mutant, for the remaining genes, a difference in direction of regulation between the protein and its transcript was observed (Figure 3; Table 2). The outcome of the combined analysis of the transcriptomic and proteomic data implies that a significant level of translational and/or post-translational regulation must have been taking place, presumably triggered by the CdS QD treatment. Moreover, they differed in their response to the treatment, in fact there was general protein requirement to be met in order to achieve resistance to CdS QDs that both mutants should achieve.

The use of plants as test organism to investigate the environmental and biological effect of ENM exposure, coupled with the exploitation of tolerant (or hypersensitive) mutants, provides a convenient means to discriminate between non-essential and essential molecular functions. The substantial number of concurrent transcripts and proteins which were regulated by the stress treatment provides the necessary sequence information which can be used in risk assessment through the construction of exposition and effect markers.

### Conflict of interest statement

The authors declare that the research was conducted in the absence of any commercial or financial relationships that could be construed as a potential conflict of interest.
