# Supplementary material for: The Proteomic Response of Arabidopsis thaliana to Cadmium Sulfide Quantum Dots, and Its Correlation with the Transcriptomic Response
Source: Front Plant Sci. 2015 Dec 16;6:1104. doi: 10.3389/fpls.2015.01104 (PMC4679877; doi:10.3389/fpls.2015.01104)
Supplement: Supplementary file 1 [file DataSheet1.PDF]

SUPPLEMENTARY MATERIAL

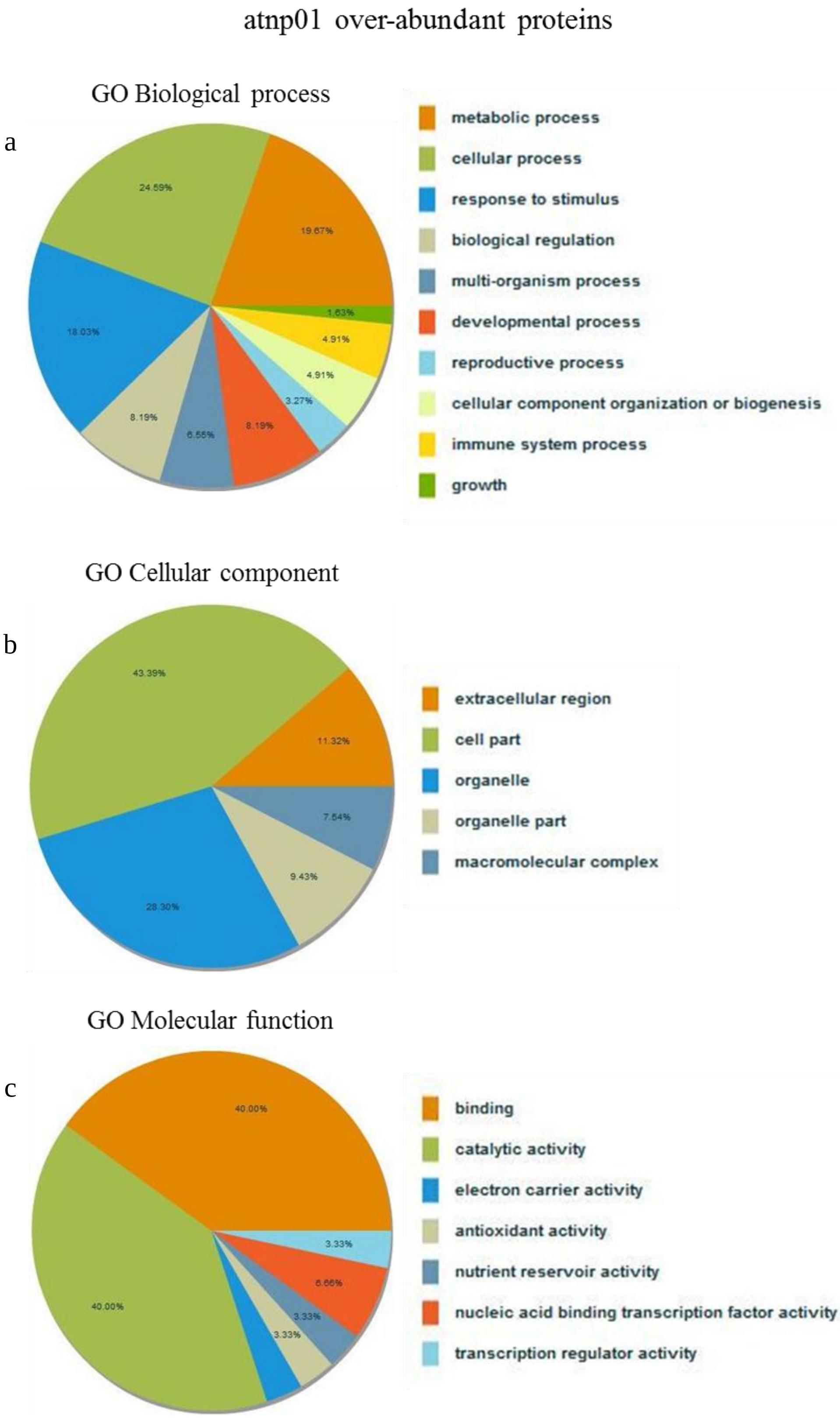

Figure S1: Pie chart analysis illustrating the classes of over-represented proteins during CdS QDs stress in atnp01 mutant line. The diagrams evidence with different colors according to the legend the percentage of the over-represented proteins. (a): over-represented proteins grouped on the basis of GO Biological processes in which they are involved. (b): over-represented proteins grouped on the basis of their GO Cellular localization. (c): over-represented proteins grouped on the basis of their GO Molecular function.

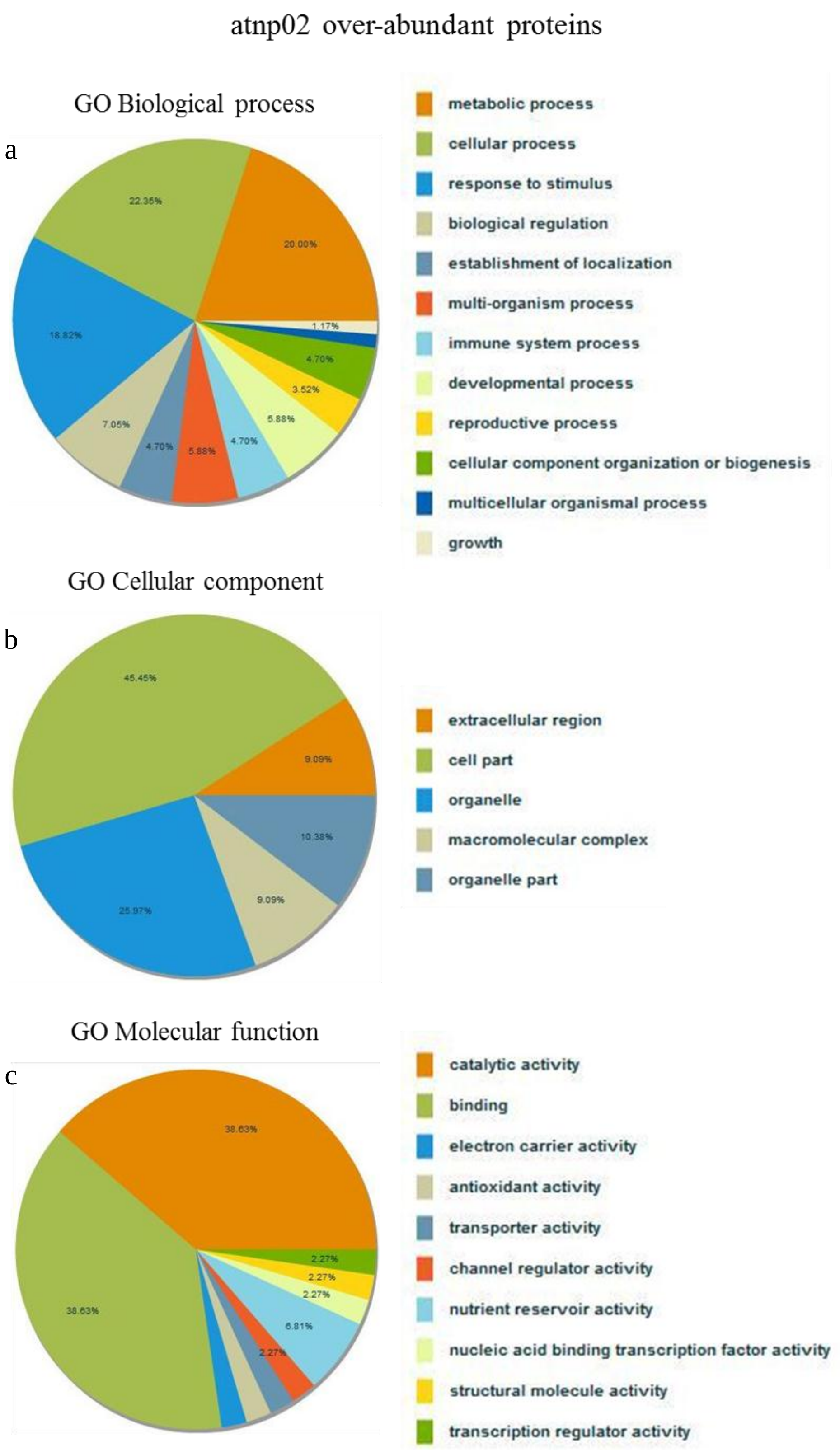

Figure S2: Pie chart analysis illustrating the classes of over-represented proteins during CdS QDs stress in atnp02 mutant line. The diagrams evidence with different colors according to the legend the percentage of the under- represented proteins. (a): under-represented proteins grouped on the basis of GO Biological processes in which they are involved. (b): under-represented proteins grouped on the basis of their GO Cellular localization. (c): under-represented proteins grouped on the basis of their GO Molecular function.

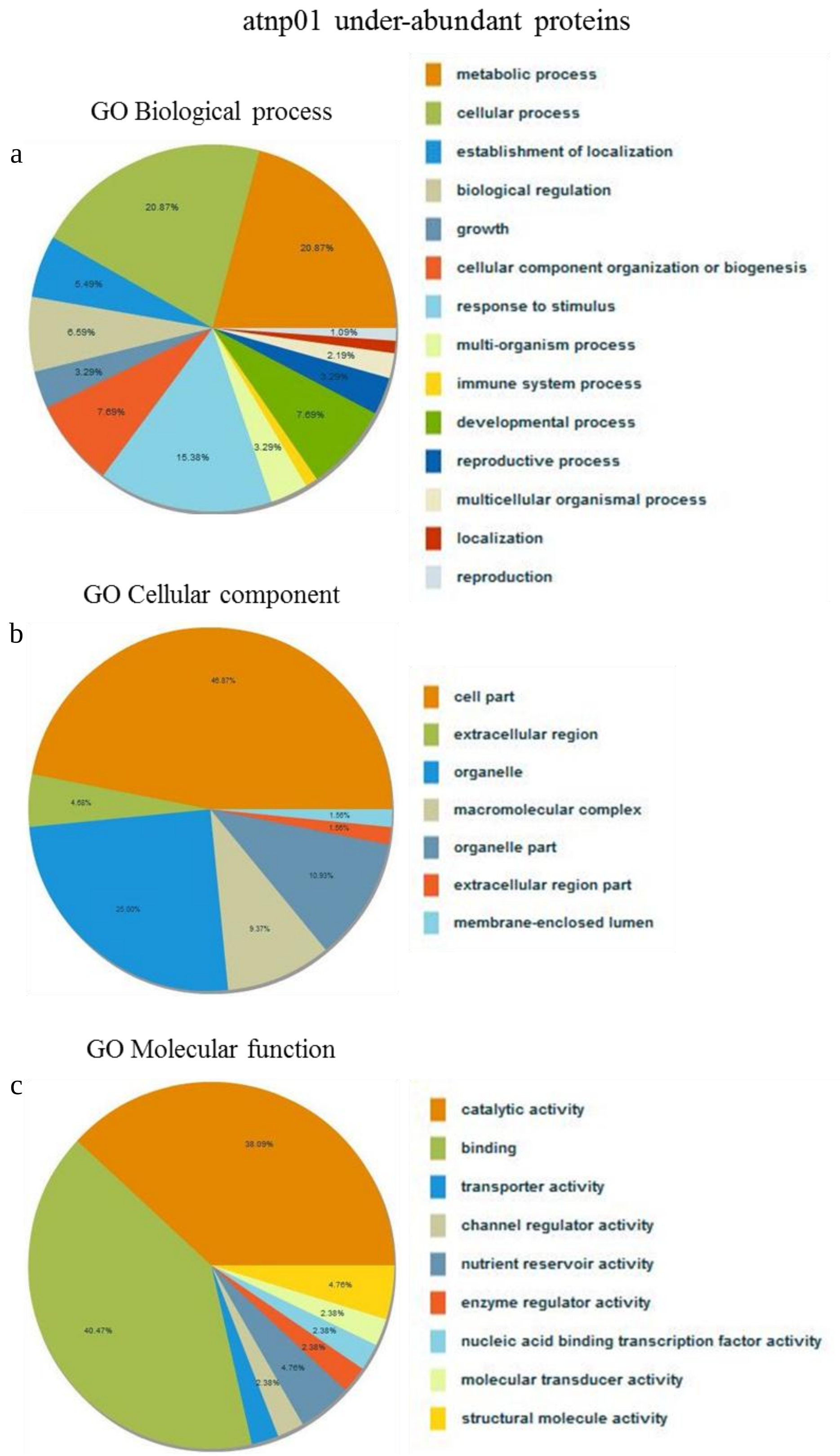

Figure S3: Pie chart analysis illustrating the classes of under-represented proteins during CdS QDs stress in atnp01 mutant line. The diagrams evidence with different colors according to the legend the percentage of the over- represented proteins. (a): over-represented proteins grouped on the basis of GO Biological processes in which they are involved. (b): over-represented proteins grouped on the basis of their GO Cellular localization. (c): over-represented proteins grouped on the basis of their GO Molecular function.

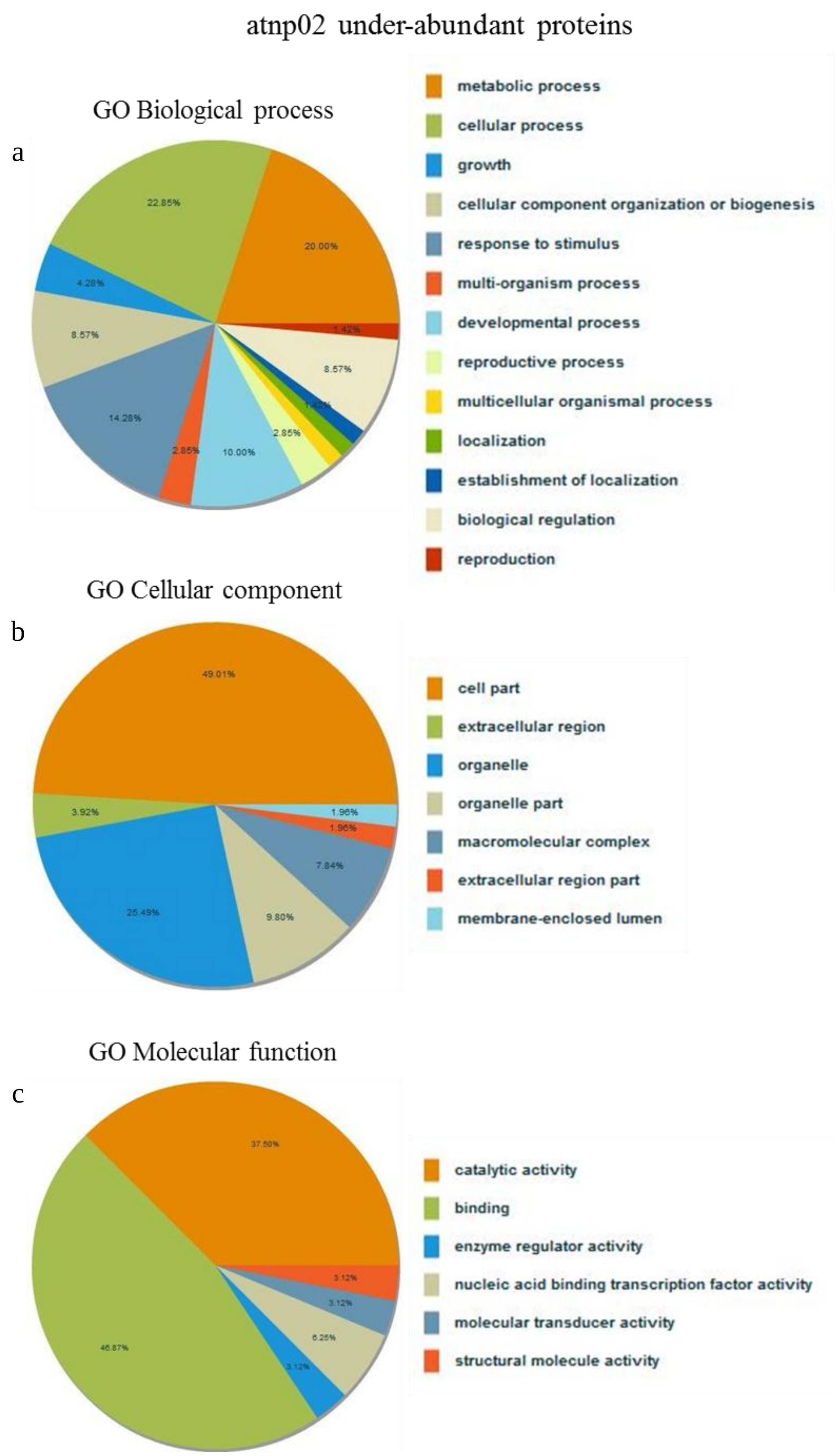

Figure S4: Pie chart analysis illustrating the classes of under-represented proteins during CdS QDs stress in atnp02 mutant line. The diagrams evidence with different colors according to the legend the percentage of the under- represented proteins. (a): under-represented proteins grouped on the basis of GO Biological processes in which they are involved. (b): under-represented proteins grouped on the basis of their GO Cellular localization. (c): under-represented proteins grouped on the basis of their GO Molecular function.

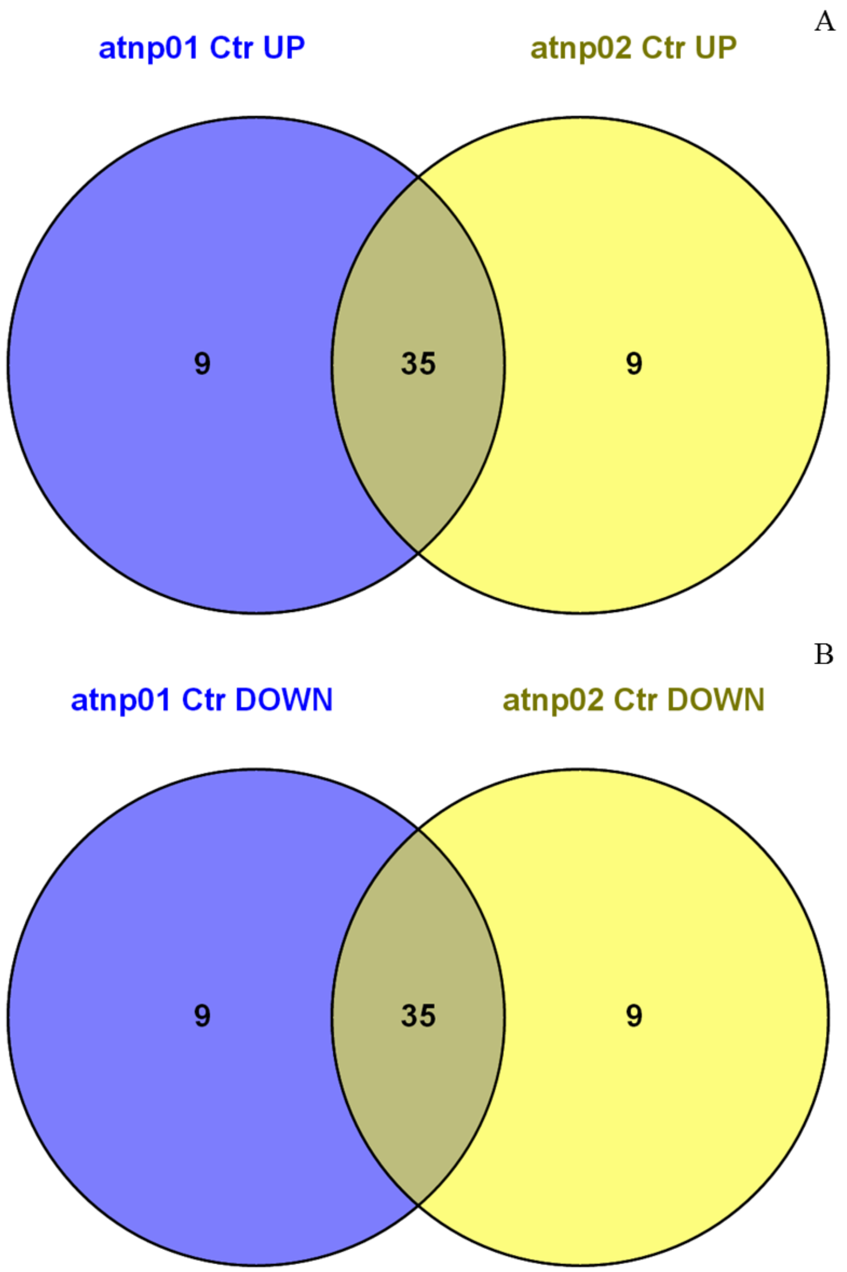

Figure S5: Venn diagrams for: (A) over-represented proteins in atnp01 and atnp02 mutant lines for control conditions, (B) under-represented proteins in atnp01 and atnp02 mutant lines for control conditions.

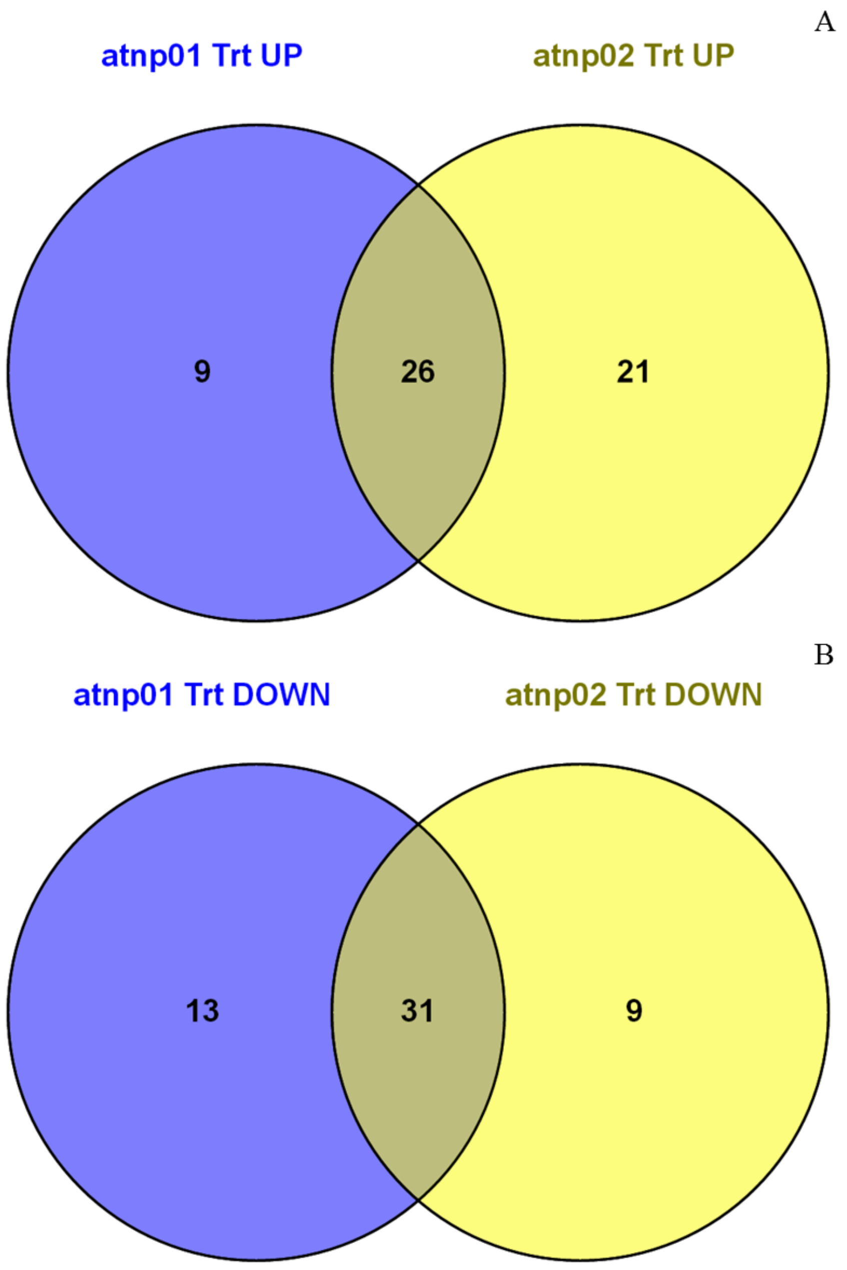

Figure S6: Venn diagrams for: (A) over-represented proteins in atnp01 and atnp02 mutant lines in treated conditions, (B) under-represented proteins in atnp01 and atnp02 mutant lines in treated conditions.

TABLE S1: over-represented proteins in atnp01 and atnp02 and relevant references. CTR: control, TRT: treated.

7 common elements in "atnp01 TRT", "atnp01 CTR", "atnp02 CTR" and "atnp02 TRT"				
Locus	Protein name (UniProt database)	Accession no.	Gene	References
At4g28520	12S seed storage protein CRC	CRU3_ARATH	CRU3	(Hegedus et al., 2015)
At4g04740	Calcium-dependent protein kinase 23	CDPKN_ARATH	CPK23	(Ma and Wu, 2007)
At3g62960	Glutaredoxin-C14	GRC14_ARATH	GRXC14	(Rouhier et al., 2006)
At5g16710	Glutathione S-transferase DHAR3	DHAR3_ARATH	DHAR3	(Sappl et al., 2004)
At2g35120	Glycine cleavage system H protein 2	GCSH2_ARATH	GDH2	(Douce et al., 2001)
At1g65660	Pre-mRNA-splicing factor SLU7-A	SLU7A_ARATH	At1g65660	(Ding et al., 2013)
At1g67090	Ribulose bisphosphate carboxylase small chain 1A	RBS1A_ARATH	RBCS-1A	(Izumi et al., 2012)
8 common elements in "atnp01 TRT" and "atnp02 TRT":				
Locus	Protein name (UniProt database)	Accession no.	Gene	
At4g17090	Beta-amylase 3, chloroplastic	BAM3_ARATH	BAM3	(Monroe et al., 2014)
At2g23980	Cyclic nucleotide-gated ion channel 6 (Probable)	CNGC6_ARATH	CNGC6	(Wang et al., 2013)
At3g51160	GDP-mannose 4,6 dehydratase 2	GMD2_ARATH	MUR1	(Bonin et al., 2003)
At1g36000	LOB domain-containing protein 5	LBD5_ARATH	LBD5	(Matsumura et al., 2009)
At3g57430	Pentatricopeptide repeat-containing protein At3g57430	PP285_ARATH	PCMP-H81	(Hammani et al., 2009)
At1g78060	Probable beta-D-xylosidase 7	BXL7_ARATH	BXL7	(Goujon et al., 2003)
At3g28480	Prolyl 4-hydroxylase 7 (probable)	P4H7_ARATH	P4H7	(Estévez et al., 2006)
At3g55005	Protein TONNEAU 1b	TON1B_ARATH	TON1B	(Azimzadeh et al., 2008)
2 elements included exclusively in "atnp01 CTR":				
Locus	Protein name (UniProt database)	Accession no.	Gene	
At1g50200	Alanine--tRNA ligase	SYA_ARATH	ALATS	(Duchêne et al., 2005)
At1g07780	N-(5'-phosphoribosyl)anthranilate isomerase 1	PAI1_ARATH	PAI1	(Melquist and Bender, 2003)
4 elements included exclusively in "atnp01 TRT":				
Locus	Protein name (UniProt database)	Accession no.	Gene	
At5g39670	Calcium-binding protein CML45 (Probable)	CML45_ARATH	CML45	(Popescu et al., 2007)
At3g20997	Defensin-like protein 121 (Putative)	DF121_ARATH	LCR55	(Vanoosthuyse et al., 2001)
At1g13870	Protein KTI12 homolog	KTI12_ARATH	DLR1	(Nelissen et al., 2003)
At1g18860	WRKY transcription factor 61 (probable)	WRK61_ARATH	WRKY61	(Eulgem et al., 2000)
1 element included exclusively in "atnp02 CTR":				
Locus	Protein name (UniProt database)	Accession no.	Gene	
At1g12775	Pentatricopeptide repeat-containing protein At1g12775	PPR39_ARATH	At1g12775	(Geddy and Brown, 2007)
11 elements included exclusively in "atnp02 TRT":				
Locus	Protein name (UniProt database)	Accession no.	Gene	
At1g08250	Arogenate dehydratase/prephenate dehydratase 6	AROD6_ARATH	ADT6	(Corea et al., 2012)
At2g31770	E3 ubiquitin-protein ligase ARI9 (probable)	ARI9_ARATH	ARI9	(Mladek et al., 2003)
At1g48625	F-box/kelch-repeat protein At1g48625	FBK20_ARATH	At1g48625	(Theologis et al., 2000)
At1g78610	Mechanosensitive ion channel protein 6	MSL6_ARATH	MSL6	(Haswell et al., 2008)
At4g26680	Pentatricopeptide repeat-containing protein At4g26680	PP338_ARATH	At4g26680	(Lurin et al., 2004)
At4g21830	Peptide methionine sulfoxide reductase B7	MSRB7_ARATH	MSRB7	(Li et al., 2012)
At2g29630	Phosphomethylpyrimidine synthase	THIC_ARATH	THIC	(Coquille et al., 2013)
At1g08780	Prefoldin subunit 4 (probable)	PFD4_ARATH	AIP3	(Rodríguez-Milla and Salinas, 2009)
At2g34990	RING-H2 finger protein ATL38	ATL38_ARATH	ATL38	(Kosarev et al., 2002)
At2g34900	Transcription factor GTE1	GTE1_ARATH	GTE1	(Duque and Chua, 2003)
At5g11390	WPP domain-interacting tail-anchored protein 1	WIT1_ARATH	WIT1	(Brkljacic et al., 2009)

TABLE S2: under-represented proteins in atnp01 and atnp02 and relevant references. CTR: control, TRT: treated.

11 common elements in "atnp01 TRT", "atnp01 CTR", "atnp02 CTR" and "atnp02 TRT":				
Locus	Protein name (UniProt database)	Accession no.	Gene	References
At2g40840	4-alpha-glucanotransferase DPE2	DPE2_ARATH	DPE2	(Lütken et al., 2010)
At3g18780	Actin-2	ACT2_ARATH	ACT2	(Kandasamy et al., 2010)
At1g10930	ATP-dependent DNA helicase Q-like 4A	RQL4A_ARATH	RECQL4A	(Higgins et al., 2011)
At5g67385	BTB/POZ domain-containing protein At5g67385	Y5738_ARATH	At5g67385	(Gingerich et al., 2005)
At2g20190	CLIP-associated protein	CLASP_ARATH	CLASP	(Ambrose et al., 2007)
At4g21100	DNA damage-binding protein 1b	DDB1B_ARATH	DDB1B	(Bernhardt et al., 2010)
At3g43710	F-box/kelch-repeat protein At3g43710 (putative)	FBK72_ARATH	At3g43710	(Ambrosone et al., 2015)
At1g62670	Pentatricopeptide repeat-containing protein At1g62670	PPR91_ARATH	At1g62670	(Binder et al., 2013)
At1g63330	Pentatricopeptide repeat-containing protein At1g63330	PP101_ARATH	At1g63330	(Geddy and Brown, 2007)
At5g61400	Pentatricopeptide repeat-containing protein At5g61400	PP440_ARATH	At5g61400	(Lurin et al., 2004)
At4g24680	Protein MODIFIER OF SNC1 1	MOS1_ARATH	MOS1	(Bao et al., 2014)
17 common elements in "atnp01 TRT" and "atnp02 TRT":				
Locus	Protein name (UniProt database)	Accession no.	Gene	
At1g75310	Auxilin-like protein 1	AUL1_ARATH	AUL1	(Quesada et al., 1999)
At2g15680	Calmodulin-like protein 1	CML1_ARATH	CML1	(Chigri et al., 2012)
At4g13235	Defensin-like protein 37	DEF37_ARATH	EDA21	(Pagnussat et al., 2005)
At1g54445	Defensin-like protein 90	DEF90_ARATH	At1g54445	(Silverstein et al., 2005)
At5g66280	GDP-mannose 4,6 dehydratase 1	GMD1_ARATH	GMD1	(Bonin et al., 2003)
At1g17170	Glutathione S-transferase U24	GSTUO_ARATH	GSTU24	(Gunning et al., 2014)
At5g09590	Heat shock 70 kDa protein 10, mitochondrial	HSP7J_ARATH	HSP70-10	(Leaden et al., 2014)
At1g62830	Lysine-specific histone demethylase 1	LDL1_ARATH	LDL1	(Zhao et al., 2015)
At3g01460	Methyl-CpG-binding domain-containing protein 9	MBD9_ARATH	MBD9	(Yaish et al., 2009)
At5g05660	NF-X1-type zinc finger protein NFXL2	NFXL2_ARATH	NFXL2	(Lisso et al., 2012)
At5g07530	Oleosin GRP-17	GRP17_ARATH	GRP17	(Mayfield and Preuss, 2000)
At5g42790	Proteasome subunit alpha type-1-A	PSA1A_ARATH	PAF1	(Sung et al., 2009)
At1g67170	Protein FLX-like 2	FLXL2_ARATH	FLXL2	(Panjabi et al., 2008)
At5g57380	Protein VERNALIZATION INSENSITIVE 3	VIN3_ARATH	VIN3	(Lee et al., 2015)
AtCg00490	Ribulose bisphosphate carboxylase large chain	RBL_ARATH	rbcL	(He et al., 2015)
At4g38430	Rop guanine nucleotide exchange factor 1	ROGF1_ARATH	ROPGEF1	(Li and Liu, 2012)
At4g12960	γ-interferon responsive lysosomal thiol (GILT) reductase family protein	F4JRI7_ARATH	At4g12960	(Wellmer et al., 2004)
6 elements included exclusively in "atnp01 CTR":				
Locus	Protein name (UniProt database)	Accession no.	Gene	
At2g36180	Calcium-binding protein CML31 (probable)	CML31_ARATH	CML31	(Lee et al., 2005)
At2g18940	Pentatricopeptide repeat-containing protein At2g18940	PP163_ARATH	At2g18940	(Ascencio-Ibáñez et al., 2008)
At2g45350	Pentatricopeptide repeat-containing protein At2g45350	PP202_ARATH	CRR4	(Boussardon et al., 2012)
At3g29290	Pentatricopeptide repeat-containing protein At3g29290	PP262_ARATH	EMB2076	(Jadhav et al., 2015)
At2g27020	Proteasome subunit alpha type-3	PSA3_ARATH	PAG1	(Book et al., 2010)
At5g36780	Proton pump-interactor 3A	PPI3A_ARATH	PPI3A	(Morandini et al., 2002)
8 elements included exclusively in "atnp01 TRT":				
Locus	Protein name (UniProt database)	Accession no.	Gene	
At4g27140	2S seed storage protein 1	2SS1_ARATH	AT2S1	(Higashi et al., 2006)
AtCg00480	ATP synthase subunit beta, chloroplastic	ATPB_ARATH	atpB	(Tan et al., 2010)
At3g02260	Auxin transport protein BIG	BIG_ARATH	BIG	(Guo et al., 2013)
At2g46480	Galacturonosyltransferase 2 (putative)	GAUT2_ARATH	GAUT2	(Sterling et al., 2006)
At1g73540	Nudix hydrolase 21	NUD21_ARATH	NUDT21	(Ogawa et al., 2008)

At2g27380	Proline-rich extensin-like protein EPR1	EPR1_ARATH	EPR1	(Dubreucq et al., 2000)
At5g38420	Ribulose bisphosphate carboxylase small chain 2B	RBS2B_ARATH	RBCS-2B	(Izumi et al., 2012)
At5g38410	Ribulose bisphosphate carboxylase small chain 3B	RBS3B_ARATH	RBCS-3B	(Zhan et al., 2014)
5 elements included exclusively in "atnp02 CTR":				
Locus	Protein name (UniProt database)	Accession no.	Gene	
At4g20780	Calcium-binding protein CML42	CML42_ARATH	CML42	(Vadassery et al., 2012)
At3g57260	Glucan endo-1,3-beta-glucosidase	E13A_ARATH	BGL2	(Wei et al., 2015)
At2g46680	Homeobox-leucine zipper protein ATHB-7	ATHB7_ARATH	ATHB-7	(Zhou et al., 2015)
At2g14610	Pathogenesis-related protein 1	PR1_ARATH	At2g14610	(Kawagoe et al., 2015)
At1g75040	Pathogenesis-related protein 5	PR5_ARATH	At1g75040	(Liu et al., 2013)
3 elements included exclusively in "atnp02 TRT":				
Locus	Protein name (UniProt database)	Accession no.	Gene	
At3g14210	GDSL esterase/lipase ESM1	ESM1_ARATH	ESM1	(Burow et al., 2008)
At3g24230	Pectate lyase 9 (probable)	PLY9_ARATH	At3g24230	(Sun and van Nocker, 2010)
At1g62590	Pentatricopeptide repeat-containing protein At1g62590	PPR90_ARATH	At1g62590	(Ruzvidzo et al., 2013)

References

Ambrose, J. C., Shoji, T., Kotzer, A. M., Pighin, J. a, and Wasteneys, G. O. (2007). The Arabidopsis CLASP gene encodes a microtubule-associated protein involved in cell expansion and division. *Plant Cell* 19, 2763–2775. doi:10.1105/tpc.107.053777.

Ambrosone, A., Batelli, G., Nurcato, R., Aurilia, V., Punzo, P., Bangarusamy, D. K., Ruberti, I., Sassi, M., Leone, A., Costa, A., et al. (2015). The Arabidopsis AtRGGA RNA binding protein regulates tolerance to salt and drought stress. *Plant Physiol.*, pp.114.255802. doi:10.1104/pp.114.255802.

Ascencio-Ibáñez, J. T., Sozzani, R., Lee, T.-J., Chu, T.-M., Wolfinger, R. D., Cella, R., and Hanley-Bowdoin, L. (2008). Global analysis of Arabidopsis gene expression uncovers a complex array of changes impacting pathogen response and cell cycle during geminivirus infection. *Plant Physiol.* 148, 436–454. doi:10.1104/pp.108.121038.

Azimzadeh, J., Nacry, P., Christodoulidou, A., Drevensek, S., Camilleri, C., Amiour, N., Parcy, F., Pastuglia, M., and Bouchez, D. (2008). Arabidopsis TONNEAU1 proteins are essential for preprophase band formation and interact with centrin. *Plant Cell* 20, 2146–2159. doi:10.1105/tpc.107.056812.

Bao, Z., Zhang, N., and Hua, J. (2014). Endopolyploidization and flowering time are antagonistically regulated by checkpoint component MAD1 and immunity modulator MOS1. *Nat. Commun.* 5, 1–10. doi:10.1038/ncomms6628.

Bernhardt, A., Mooney, S., and Hellmann, H. (2010). Arabidopsis DDB1a and DDB1b are critical for embryo development. *Planta* 232, 555–566. doi:10.1007/s00425-010-1195-9.

Binder, S., Stoll, K., and Stoll, B. (2013). P-class pentatricopeptide repeat proteins are required for efficient 5’ end formation of plant mitochondrial transcripts. *RNA Biol.* 10, 1511–9. doi:10.4161/rna.26129.

Bonin, C. P., Freshour, G., Hahn, M. G., Vanzin, G. F., and Reiter, W. (2003). The GMD1 and GMD2 Genes of Arabidopsis Encode Isoforms of GDP- D -Mannose 4 , 6-Dehydratase with

Cell Type-Specific Expression Patterns 1. *Society* 132, 883–892. doi:10.1104/pp.103.022368.which.

Book, A. J., Gladman, N. P., Lee, S. S., Scalf, M., Smith, L. M., and Vierstra, R. D. (2010). Affinity purification of the Arabidopsis 26 S proteasome reveals a diverse array of plant proteolytic complexes. *J. Biol. Chem.* 285, 25554–25569. doi:10.1074/jbc.M110.136622.

Boussardon, C., Salone, V., Avon, a., Berthome, R., Hammani, K., Okuda, K., Shikanai, T., Small, I., and Lurin, C. (2012). Two Interacting Proteins Are Necessary for the Editing of the NdhD-1 Site in Arabidopsis Plastids. *Plant Cell* 24, 3684–3694. doi:10.1105/tpc.112.099507.

Brkljacic, J., Zhao, Q., and Meier, I. (2009). WPP-domain proteins mimic the activity of the HSC70-1 chaperone in preventing mistargeting of RanGAP1-anchoring protein WIT1. *Plant Physiol.* 151, 142–154. doi:10.1104/pp.109.143404.

Burow, M., Zhang, Z. Y., Ober, J. a., Lambrix, V. M., Wittstock, U., Gershenzon, J., and Kliebenstein, D. J. (2008). ESP and ESM1 mediate indol-3-acetonitrile production from indol-3-ylmethyl glucosinolate in Arabidopsis. *Phytochemistry* 69, 663–671. doi:10.1016/j.phytochem.2007.08.027.

Chigri, F., Flosdorff, S., Pilz, S., Kölle, E., Dolze, E., Gietl, C., and Vothknecht, U. C. (2012). The Arabidopsis calmodulin-like proteins AtCML30 and AtCML3 are targeted to mitochondria and peroxisomes, respectively. *Plant Mol. Biol.* 78, 211–222. doi:10.1007/s11103-011-9856-z.

Coquille, S., Roux, C., Mehta, A., Begley, T. P., Fitzpatrick, T. B., and Thore, S. (2013). High-resolution crystal structure of the eukaryotic HMP-P synthase (THIC) from Arabidopsis thaliana. *J. Struct. Biol.* 184, 438–444. doi:10.1016/j.jsb.2013.10.005.

Corea, O. R. a, Ki, C., Cardenas, C. L., Kim, S. J., Brewer, S. E., Patten, A. M., Davin, L. B., and Lewis, N. G. (2012). Arogenate dehydratase isoenzymes profoundly and differentially modulate carbon flux into lignins. *J. Biol. Chem.* 287, 11446–11459. doi:10.1074/jbc.M111.322164.

Ding, Y., Liu, N., Virlouvet, L., Riethoven, J.-J., Fromm, M., and Avramova, Z. (2013). Four distinct types of dehydration stress memory genes in Arabidopsis thaliana. *BMC Plant Biol.* 13, 229. doi:10.1186/1471-2229-13-229.

Douce, R., Bourguignon, J., Neuburger, M., and Rébeillé, F. (2001). The glycine decarboxylase system: A fascinating complex. *Trends Plant Sci.* 6, 167–176. doi:10.1016/S1360-1385(01)01892-1.

Dubreucq, B., Berger, N., Vincent, E., Boisson, M., Pelletier, G., Caboche, M., and Lepiniec, L. (2000). The Arabidopsis AtEPR1 extensin-like gene is specifically expressed in endosperm during seed germination. *Plant J.* 23, 643–652. doi:10.1046/j.1365-313X.2000.00829.x.

Duchêne, A.-M., Giritch, A., Hoffmann, B., Cognat, V., Lancelin, D., Peeters, N. M., Zaepfel, M., Maréchal-Drouard, L., and Small, I. D. (2005). Dual targeting is the rule for organellar aminoacyl-tRNA synthetases in Arabidopsis thaliana. *Proc. Natl. Acad. Sci. U. S. A.* 102, 16484–16489. doi:10.1073/pnas.0504682102.

Duque, P., and Chua, N. H. (2003). IMB1, a bromodomain protein induced during seed imbibition, regulates ABA- and phyA-mediated responses of germination in Arabidopsis. *Plant J.* 35, 787–799. doi:10.1046/j.1365-313X.2003.01848.x.

Estévez, J. M., Kieliszewski, M. J., Khitrov, N., and Somerville, C. (2006). Characterization of synthetic hydroxyproline-rich proteoglycans with arabinogalactan protein and extensin motifs in Arabidopsis. *Plant Physiol.* 142, 458–470. doi:10.1104/pp.106.084244.

Eulgem, T., Rushton, P. J., Robatzek, S., and Somssich, I. E. (2000). The WRKY superfamily of plant transcription factors. *Trends Plant Sci.* 5, 199–206. doi:10.1016/S1360-1385(00)01600-9.

Geddy, R., and Brown, G. G. (2007). Genes encoding pentatricopeptide repeat (PPR) proteins are not conserved in location in plant genomes and may be subject to diversifying selection. *BMC Genomics* 8, 130. doi:10.1186/1471-2164-8-130.

Gingerich, D. J., Gagne, J. M., Salter, D. W., Hellmann, H., Estelle, M., Ma, L., and Vierstra, R. D. (2005). Cullins 3a and 3b assemble with members of the broad complex/tramtrack/ bric-a-brac (BTB) protein family to form essential ubiquitin-protein ligases (E3s) in arabidopsis. *J. Biol. Chem.* 280, 18810–18821. doi:10.1074/jbc.M413247200.

Goujon, T., Minic, Z., El Amrani, A., Lerouxel, O., Aletti, E., Lapierre, C., Joseleau, J. P., and Jouanin, L. (2003). AtBXL1, a novel higher plant (Arabidopsis thaliana) putative beta-xylosidase gene, is involved in secondary cell wall metabolism and plant development. *Plant J.* 33, 677–690. doi:10.1046/j.1365-313X.2003.01654.x.

Gunning, V., Tzafestas, K., Sparrow, H., Johnston, E. J., Brentnall, A. S., Potts, J. R., Rylott, E. L., and Bruce, N. C. (2014). Arabidopsis Glutathione Transferases U24 and U25 Exhibit a Range of Detoxification Activities with the Environmental Pollutant and Explosive, 2,4,6-Trinitrotoluene. *Plant Physiol.* 165, 854–865. doi:10.1104/pp.114.237180.

Guo, X., Lu, W., Ma, Y., Qin, Q., and Hou, S. (2013). The BIG gene is required for auxin-mediated organ growth in Arabidopsis. *Planta* 237, 1135–1147. doi:10.1007/s00425-012-1834-4.

Hammani, K., Okuda, K., Tanz, S. K., Chateigner-Boutin, A.-L., Shikanai, T., and Small, I. (2009). A study of new Arabidopsis chloroplast RNA editing mutants reveals general features of editing factors and their target sites. *Plant Cell* 21, 3686–3699. doi:10.1105/tpc.109.071472.

Haswell, E. S., Peyronnet, R., Barbier-Brygoo, H., Meyerowitz, E. M., and Frachisse, J. M. (2008). Two MscS Homologs Provide Mechanosensitive Channel Activities in the Arabidopsis Root. *Curr. Biol.* 18, 730–734. doi:10.1016/j.cub.2008.04.039.

He, B., Mu, Y., and Chi, W. (2015). Effects of inefficient transcription termination of rbcL on the expression of accD in plastids of Arabidopsis thaliana. *Photosynth. Res.* doi:10.1007/s11120-015-0159-0.

Hegedus, D. D., Coutu, C., Harrington, M., Hope, B., Gerbrandt, K., and Nikolov, I. (2015). Multiple internal sorting determinants can contribute to the trafficking of cruciferin to protein storage vacuoles. *Plant Mol. Biol.* 88, 3–20. doi:10.1007/s11103-015-0297-y.

Higashi, Y., Hirai, M. Y., Fujiwara, T., Naito, S., Noji, M., and Saito, K. (2006). Proteomic and transcriptomic analysis of Arabidopsis seeds: Molecular evidence for successive processing of seed proteins and its implication in the stress response to sulfur nutrition. *Plant J.* 48, 557–571. doi:10.1111/j.1365-313X.2006.02900.x.

Higgins, J. D., Ferdous, M., Osman, K., and Franklin, F. C. H. (2011). The RecQ helicase AtRECQ4A is required to remove inter-chromosomal telomeric connections that arise during meiotic recombination in Arabidopsis. *Plant J.* 65, 492–502. doi:10.1111/j.1365-313X.2010.04438.x.

Izumi, M., Tsunoda, H., Suzuki, Y., Makino, A., and Ishida, H. (2012). RBCS1A and RBCS3B, two major members within the Arabidopsis RBCS multigene family, function to yield sufficient Rubisco content for leaf photosynthetic capacity. *J. Exp. Bot.* 63, 2159–2170. doi:10.1093/jxb/err434.

Jadhav, A. A., Rayate, S. J., Mhase, L. B., Thudi, M., Chitikineni, A., and Harer, P. N. (2015). Marker-trait association study for protein content in chickpea (Cicer arietinum L .). 94, 279–286.

Kandasamy, M. K., McKinney, E. C., and Meagher, R. B. (2010). Differential sublocalization of actin variants within the nucleus. *Cytoskeleton* 67, 729–743. doi:10.1002/cm.20484.

Kawagoe, Y., Shiraishi, S., Kondo, H., Yamamoto, S., Aoki, Y., and Suzuki, S. (2015). Cyclic lipopeptide iturin A structure-dependently induces defense response in Arabidopsis plants by activating SA and JA signaling pathways. *Biochem. Biophys. Res. Commun.* 460, 1015–1020. doi:10.1016/j.bbrc.2015.03.143.

Kosarev, P., Mayer, K. F. X., and Hardtke, C. S. (2002). Evaluation and classification of RING-finger domains encoded by the Arabidopsis genome. *Genome Biol.* 3, RESEARCH0016.

Leaden, L., Busi, M. V., and Gomez-Casati, D. F. (2014). The mitochondrial proteins AtHscB and AtIsu1 involved in Fe–S cluster assembly interact with the Hsp70-type chaperon AtHscA2 and modulate its catalytic activity. *Mitochondrion* 19, 375–381. doi:10.1016/j.mito.2014.11.002.

Lee, D., Polisensky, D. H., and Braam, J. (2005). Genome-wide identification of touch- and darkness-regulated Arabidopsis genes: A focus on calmodulin-like and XTH genes. *New Phytol.* 165, 429–444. doi:10.1111/j.1469-8137.2004.01238.x.

Lee, J., Yun, J.-Y., Zhao, W., Shen, W.-H., and Amasino, R. M. (2015). A methyltransferase required for proper timing of the vernalization response in Arabidopsis. *Proc. Natl. Acad. Sci.*, 201423585. doi:10.1073/pnas.1423585112.

Li, C. W., Lee, S. H., Chieh, P. S., Lin, C. S., Wang, Y. C., and Chan, M. T. (2012). Arabidopsis Root-Abundant Cytosolic Methionine Sulfoxide Reductase B Genes MsrB7 and MsrB8 are Involved in Tolerance to Oxidative Stress. *Plant Cell Physiol.* 53, 1707–1719. doi:10.1093/pcp/pcs114.

Li, Z., and Liu, D. (2012). ROPGEF1 and ROPGEF4 are functional regulators of ROP11 GTPase in ABA-mediated stomatal closure in Arabidopsis. *FEBS Lett.* 586, 1253–1258. doi:10.1016/j.febslet.2012.03.040.

Lisso, J., Schröder, F., Schippers, J. H. M., and Müssig, C. (2012). NFXL2 modifies cuticle properties in Arabidopsis. *Plant Signal. Behav.* 7, 551–555. doi:10.4161/psb.19838.

Liu, W. X., Zhang, F. C., Zhang, W. Z., Song, L. F., Wu, W. H., and Chen, Y. F. (2013). Arabidopsis Di19 functions as a transcription factor and modulates PR1, PR2, and PR5 expression in response to drought stress. *Mol. Plant* 6, 1487–1502. doi:10.1093/mp/sst031.

Lurin, C., Andrés, C., Aubourg, S., Bellaoui, M., Bitton, F., Bruyère, C., Caboche, M., Debast, C., Gualberto, J., Hoffmann, B., et al. (2004). Genome-wide analysis of Arabidopsis pentatricopeptide repeat proteins reveals their essential role in organelle biogenesis. *Plant Cell* 16, 2089–2103. doi:10.1105/tpc.104.022236.

Lütken, H., Lloyd, J. R., Glaring, M. a., Baunsgaard, L., Laursen, K. H., Haldrup, A., Kossmann, J., and Blennow, A. (2010). Repression of both isoforms of disproportionating enzyme leads to higher malto-oligosaccharide content and reduced growth in potato. *Planta* 232, 1127–1139. doi:10.1007/s00425-010-1245-3.

Ma, S. Y., and Wu, W. H. (2007). AtCPK23 functions in Arabidopsis responses to drought and salt stresses. *Plant Mol. Biol.* 65, 511–518. doi:10.1007/s11103-007-9187-2.

Matsumura, Y., Iwakawa, H., MacHida, Y., and MacHida, C. (2009). Characterization of genes in the ASYMMETRIC LEAVES2-LATERAL ORGAN BOUNDARIES (AS2-LOB) family in Arabidopsis thaliana, and functional and molecular comparisons between AS2 and other family members. *Plant J.* 58, 525–537. doi:10.1111/j.1365-313X.2009.03797.x.

Mayfield, J. a, and Preuss, D. (2000). Rapid initiation of Arabidopsis pollination requires the oleosin-domain protein GRP17. *Nat. Cell Biol.* 2, 128–130. doi:10.1038/35000084.

Melquist, S., and Bender, J. (2003). Transcription from an upstream promoter controls methylation signaling from an inverted repeat of endogenous genes in Arabidopsis. *Genes Dev.* 17, 2036–2047. doi:10.1101/gad.1081603.

Mladek, C., Mladek, C., Guger, K., Guger, K., Hauser, M., and Hauser, M. (2003). Identification and Characterization of the. *Society* 131, 27–40. doi:10.1104/pp.012781.dence.

Monroe, J. D., Storm, a. R., Badley, E. M., Lehman, M. D., Platt, S. M., Saunders, L. K., Schmitz, J. M., and Torres, C. E. (2014). -Amylase1 and -Amylase3 Are Plastidic Starch Hydrolases in Arabidopsis That Seem to Be Adapted for Different Thermal, pH, and Stress Conditions. *Plant Physiol.* 166, 1748–1763. doi:10.1104/pp.114.246421.

Morandini, P., Valera, M., Albumi, C., Bonza, M. C., Giacometti, S., Ravera, G., Murgia, I., Soave, C., and De Michelis, M. I. (2002). A novel interaction partner for the C-terminus of Arabidopsis thaliana plasma membrane H+-ATPase (AHA1 isoform): Site and mechanism of action on H+-ATPase activity differ from those of 14-3-3 proteins. *Plant J.* 31, 487–497. doi:10.1046/j.1365-313X.2002.01373.x.

Nelissen, H., Clarke, J. H., De Block, M., De Block, S., Vanderhaeghen, R., Zielinski, R. E., Dyer, T., Lust, S., Inzé, D., and Van Lijsebettens, M. (2003). DRL1, a homolog of the yeast TOT4/KTI12 protein, has a function in meristem activity and organ growth in plants. *Plant Cell* 15, 639–654. doi:10.1105/tpc.007062.

Ogawa, T., Yoshimura, K., Miyake, H., Ishikawa, K., Ito, D., Tanabe, N., and Shigeoka, S. (2008). Molecular characterization of organelle-type Nudix hydrolases in Arabidopsis. *Plant Physiol.* 148, 1412–1424. doi:10.1104/pp.108.128413.

Pagnussat, G. C., Yu, H.-J., Ngo, Q. a, Rajani, S., Mayalagu, S., Johnson, C. S., Capron, A., Xie, L.-F., Ye, D., and Sundaresan, V. (2005). Genetic and molecular identification of genes required for female gametophyte development and function in Arabidopsis. *Development* 132, 603–614. doi:10.1242/dev.01595.

Panjabi, P., Jagannath, A., Bisht, N. C., Padmaja, K. L., Sharma, S., Gupta, V., Pradhan, A. K., and Pental, D. (2008). Comparative mapping of Brassica juncea and Arabidopsis thaliana using Intron Polymorphism (IP) markers: homoeologous relationships, diversification and evolution of the A, B and C Brassica genomes. *BMC Genomics* 9, 113. doi:10.1186/1471-2164-9-113.

Popescu, S. C., Popescu, G. V, Bachan, S., Zhang, Z., Seay, M., Gerstein, M., Snyder, M., and Dinesh-Kumar, S. P. (2007). Differential binding of calmodulin-related proteins to their targets revealed through high-density Arabidopsis protein microarrays. *Proc. Natl. Acad. Sci. U. S. A.* 104, 4730–4735. doi:10.1073/pnas.0611615104.

Quesada, V., Ponce, M. R., and Micol, J. L. (1999). OTC and AUL1, two convergent and overlapping genes in the nuclear genome of Arabidopsis thaliana. *FEBS Lett.* 461, 101–106. doi:10.1016/S0014-5793(99)01426-X.

Rodríguez-Milla, M. a., and Salinas, J. (2009). Prefoldins 3 and 5 play an essential role in Arabidopsis tolerance to salt stress. *Mol. Plant* 2, 526–534. doi:10.1093/mp/ssp016.

Rouhier, N., Couturier, J., and Jacquot, J. P. (2006). Genome-wide analysis of plant glutaredoxin systems. *J. Exp. Bot.* 57, 1685–1696. doi:10.1093/jxb/erl001.

Ruzvidzo, O., Dikobe, B. T., Kawadza, D. T., Mabadahanye, G. H., Chatukuta, P., and Kwezi, L. (2013). Recombinant Expression and Functional Testing of Candidate Adenylate Cyclase Domains. *Methods Mol. Biol.* 1016, 13–25. doi:10.1007/978-1-62703-441-8_2.

Sappl, P. G., Oñate-Sánchez, L., Singh, K. B., and Millar, a. H. (2004). Proteomic analysis of glutathione S-transferases of Arabidopsis thaliana reveals differential salicylic acid-induced expression of the plant-specific phi and tau classes. *Plant Mol. Biol.* 54, 205–219. doi:10.1023/B:PLAN.0000028786.57439.b3.

Silverstein, K. a T., Graham, M. a, Paape, T. D., and VandenBosch, K. a (2005). Genome organization of more than 300 defensin-like genes in Arabidopsis. *Plant Physiol.* 138, 600–610. doi:10.1104/pp.105.060079.

Sterling, J. D., Atmodjo, M. a, Inwood, S. E., Kumar Kolli, V. S., Quigley, H. F., Hahn, M. G., and Mohnen, D. (2006). Functional identification of an Arabidopsis pectin biosynthetic homogalacturonan galacturonosyltransferase. *Proc. Natl. Acad. Sci. U. S. A.* 103, 5236–5241. doi:10.1073/pnas.0600120103.

Sun, L., and van Nocker, S. (2010). Analysis of promoter activity of members of the PECTATE LYASE-LIKE (PLL) gene family in cell separation in Arabidopsis. *BMC Plant Biol.* 10, 152. doi:10.1186/1471-2229-10-152.

Sung, D. Y., Kim, T. H., Komives, E. a., Mendoza-Cózatl, D. G., and Schroeder, J. I. (2009). ARS5 is a component of the 26S proteasome complex, and negatively regulates thiol biosynthesis and arsenic tolerance in Arabidopsis. *Plant J.* 59, 802–812. doi:10.1111/j.1365-313X.2009.03914.x.

Tan, Y.-F., O'Toole, N., Taylor, N. L., and Millar, a H. (2010). Divalent metal ions in plant mitochondria and their role in interactions with proteins and oxidative stress-induced damage to respiratory function. *Plant Physiol.* 152, 747–761. doi:10.1104/pp.109.147942.

Theologis, A., Ecker, J. R., Palmk, C. J., Federspiel, N. a, Kaul, S., White, O., Alonso, J., Alta, H., Araujok, R., Bowman, C. L., et al. (2000). Sequence and analysis of chromosome 1 of the plant Arabidopsis thaliana. 408.

Vadassery, J., Reichelt, M., Hause, B., Gershenzon, J., Boland, W., and Mithofer, a. (2012). CML42-Mediated Calcium Signaling Coordinates Responses to Spodoptera Herbivory and Abiotic Stresses in Arabidopsis. *Plant Physiol.* 159, 1159–1175. doi:10.1104/pp.112.198150.

Vanoosthuyse, V., Miege, C., Dumas, C., and Cock, J. M. (2001). Two large Arabidopsis thaliana gene families are homologous to the Brassica gene superfamily that encodes pollen coat proteins and the male component of the self-incompatibility response. *Plant Mol. Biol.* 46, 17–34. doi:10.1023/A:1010664704926.

Wang, Y.-F., Munemasa, S., Nishimura, N., Ren, H.-M., Robert, N., Han, M., Puzõrjova, I., Kollist, H., Lee, S., Mori, I., et al. (2013). Identification of cyclic GMP-activated nonselective Ca2+-permeable cation channels and associated CNGC5 and CNGC6 genes in Arabidopsis guard cells. *Plant Physiol.* 163, 578–90. doi:10.1104/pp.113.225045.

Wei, H., Brunecky, R., Donohoe, B. S., Ding, S.-Y., Ciesielski, P. N., Yang, S., Tucker, M. P., and Himmel, M. E. (2015). Identifying the ionically bound cell wall and intracellular glycoside hydrolases in late growth stage Arabidopsis stems: implications for the genetic engineering of bioenergy crops. *Front. Plant Sci.* 6. doi:10.3389/fpls.2015.00315.

Wellmer, F., Riechmann, L., Alves-Ferreira, M., and Meyerowitz, E. M. (2004). Genome-Wide Analysis of Spatial Gene Expression in Arabidopsis Flowers. *Plant Cell* 16, 1314–1326. doi:10.1105/tpc.021741.termination.

Yaish, M. W. F., Peng, M., and Rothstein, S. J. (2009). AtMBD9 modulates Arabidopsis development through the dual epigenetic pathways of DNA methylation and histone acetylation. *Plant J.* 59, 123–135. doi:10.1111/j.1365-313X.2009.03860.x.

Zhan, G.-M., Li, R.-J., Hu, Z.-Y., Liu, J., Deng, L.-B., Lu, S.-Y., and Hua, W. (2014). Cosuppression of RBCS3B in Arabidopsis leads to severe photoinhibition caused by ROS accumulation. *Plant Cell Rep.* 33, 1091–1108. doi:10.1007/s00299-014-1597-4.

Zhao, M., Yang, S., Liu, X., and Wu, K. (2015). Arabidopsis histone demethylases LDL1 and LDL2 control primary seed dormancy by regulating DELAY OF GERMINATION 1 and ABA signaling-related genes. *Front. Plant Sci.* 6, 1–9. doi:10.3389/fpls.2015.00159.

Zhou, S., Sun, L., Vald, A. E., Engstr, P., Song, Z., Lu, S., and Liu, J. (2015). Membrane-associated transcription factor peptidase , site-2 protease , antagonizes ABA signaling in Arabidopsis. 5.
